# Large scale detailed mapping of dengue vector breeding sites using street view images

**DOI:** 10.1371/journal.pntd.0007555

**Published:** 2019-07-29

**Authors:** Peter Haddawy, Poom Wettayakorn, Boonpakorn Nonthaleerak, Myat Su Yin, Anuwat Wiratsudakul, Johannes Schöning, Yongjua Laosiritaworn, Klestia Balla, Sirinut Euaungkanakul, Papichaya Quengdaeng, Kittipop Choknitipakin, Siripong Traivijitkhun, Benyarut Erawan, Thansuda Kraisang

**Affiliations:** 1 Faculty of ICT, Mahidol University, Salaya, Thailand; 2 Bremen Spatial Cognition Center, University of Bremen, Bremen, Germany; 3 Faculty of Veterinary Science, Mahidol University, Salaya, Thailand; 4 University of Bremen, Bremen, Germany; 5 Ministry of Public Health, Bangkok, Thailand; 6 Computer Science Department, School of Science and Technology, University of Camerino, Camerino, Italy; University of Washington, UNITED STATES

## Abstract

Targeted environmental and ecosystem management remain crucial in control of dengue. However, providing detailed environmental information on a large scale to effectively target dengue control efforts remains a challenge. An important piece of such information is the extent of the presence of potential dengue vector breeding sites, which consist primarily of open containers such as ceramic jars, buckets, old tires, and flowerpots. In this paper we present the design and implementation of a pipeline to detect outdoor open containers which constitute potential dengue vector breeding sites from geotagged images and to create highly detailed container density maps at unprecedented scale. We implement the approach using Google Street View images which have the advantage of broad coverage and of often being two to three years old which allows correlation analyses of container counts against historical data from manual surveys. Containers comprising eight of the most common breeding sites are detected in the images using convolutional neural network transfer learning. Over a test set of images the object recognition algorithm has an accuracy of 0.91 in terms of F-score. Container density counts are generated and displayed on a decision support dashboard. Analyses of the approach are carried out over three provinces in Thailand. The container counts obtained agree well with container counts from available manual surveys. Multi-variate linear regression relating densities of the eight container types to larval survey data shows good prediction of larval index values with an R-squared of 0.674. To delineate conditions under which the container density counts are indicative of larval counts, a number of factors affecting correlation with larval survey data are analyzed. We conclude that creation of container density maps from geotagged images is a promising approach to providing detailed risk maps at large scale.

## Author summary

Providing detailed environmental information on a large scale to effectively target dengue control efforts remains a challenge. In this paper we present the design and implementation of a pipeline to detect outdoor open containers which constitute potential dengue vector breeding sites from geotagged images and to create highly detailed container density maps at unprecedented scale. Specifically, we use convolutional neural networks to detect a variety of types of breeding site container types in Google street view images and use the container counts to create container density maps. Evaluation of the approach is carried out over three provinces in Thailand: Bangkok, Krabi, and Nakhon Si Thammarat. Our evaluation shows that the object recognition network can accurately recognize several of the most important types of containers in Thailand. The container counts obtained from the street view images agree well with container counts from available manual surveys. We further show that simple multi-linear models using container density values provide good predictions of Breteau index (number of positive containers per 100 houses inspected) values. This is the first study to present results validating container counts from image analysis against such data.

## Introduction

Dengue is considered one of the most important mosquito-borne viral diseases in the world. During the past five decades, the incidence of dengue has increased 30-fold, with a recent study estimating global incidence at 390 million cases per year [1]. Dengue is now considered endemic in more than 100 countries, with more than two thirds of the burden found in Asia. Even in Europe, an outbreak in Madeira that began in 2012 resulted in over 2,000 cases, with imported cases from travelers to Madeira detected in 13 other European countries [2].

One dengue vaccine (CYD-TDV or Dengvaxia) has now been registered in several countries. But with about 60% effectiveness and lack of approval for use in children under 9 years old, it does not provide an effective line of defense [3]. Since there is also no curative treatment for dengue, targeted environmental and ecosystem management continue to be crucial in controlling the disease.

The *Aedes aegypti* and *Aedes albopictus* mosquitoes are the primary vectors of dengue and are additionally responsible for the spread of chikungunya, Zika fever, and yellow fever [4]. The *Aedes* mosquitoes have adapted to human habitats and breed in relatively small containers that can hold water such as ceramic jars, old tires, flower pots, and buckets. Studies of the dispersal of *Aedes aegypti* and *Aedes albopictus* indicate that the mosquitoes actively disperse over only short ranges [5–7]. In an analysis of *Aedes aegypti* flight range and dispersal patterns from 21 mark-release-recapture experiments conducted over 11 years in Puerto Rico and Thailand, Harrington et al (2005) [5] found that the majority of released mosquitos were recaptured in the same house or adjacent house to where they were released. The mean dispersal distances ranged from 28 to 199 meters. These results were consistent across the different experiments, including indoor and outdoor release sites.

The combination of small-scale breeding sites and low level of mobility of the vector results in highly localized sites of disease transmission with dengue transmission and dengue vector abundance exhibiting substantial geographic variability [8]. Indeed, some studies have found spatial heterogeneity of the dengue vector at the neighborhood level [9,10]. Others have even found spatial heterogeneity of the dengue vector at the household level [11,12] and similarly for dengue transmission [13,14].

Two primary approaches have been taken to provide environmental data for dengue risk mapping and prediction. The first is to use remote sensing [15] or proxies (e.g. per capita number of public small water wells [16], number of households having a rain water tank [17], and the type of housing (individual house versus apartments, large residential area) [18]) to assess local environmental conditions [19]. Proxies provide only indirect evidence about breeding sites, and remote sensing, even from aerial photography, can be inaccurate due to canopy cover and other issues [20]. A second approach is to carry out manual surveys in which containers with water or containers with water and larvae are counted [21] [22]. Results are then reported in terms of numbers of containers of different types or in terms of larval indices: Breteau Index (number of positive containers per 100 houses inspected), House Index (percentage of houses infested with larvae and/or pupae), and Container Index (percentage of water holding containers infested with larvae or pupae) [23,24]. While this approach provides direct information about breeding sites, it is not scalable due to its labor-intensive nature. Thus, there is a need for an approach that can provide direct information on potential breeding sites at high resolution and that is scalable to cover major cities and provinces.

In this study, we address this problem by using convolutional neural networks (CNN) to detect breeding site container types in geotagged images and using the resulting container counts to create container density maps. While our architecture can accommodate geotagged images from a wide variety of sources, in this study we use Google Street View (GSV) images due to the extensive geographic coverage and the historical nature of much of the image data, which allows it to be temporally aligned with container and larval counts from previous manual surveys for evaluation. Evaluation of the approach is carried out over three provinces in Thailand: Bangkok, Krabi, and Nakhon Si Thammarat. Our evaluation shows that the object recognition network can accurately recognize several of the most important types of containers in Thailand. We provide detailed statistics on GSV image coverage and container counts at the district level. The container counts obtained from the GSV images agree well with container counts from available manual surveys. We further show that simple multi-linear models using container density values provide good predictions of Breteau index values. This is the first study to present results comparing container counts from image analysis against container and larval counts from manual surveys, providing evidence for their potential usefulness in mapping suitable conditions for vector abundance.

### Related work

In their review of dengue risk mapping modeling tools, Louis et al. [25] showed that social predictors such as education level, occupational status, and income are often used as proxies to assess local environmental conditions and hygiene, which are normally difficult to assess directly. Housing conditions are often used as a proxy to assess type and number of mosquito breeding sites. Lack of access to running water has also been found to be a risk factor for dengue since residents in such areas tend to store water in ground-level containers [26–27]. Chang et al. [28] used satellite imagery from Google Earth to create a base map to which they added information about larval infestation, locations of tire dumps, cemeteries, large areas of standing water, and locations of homes of dengue cases, all of which were collected manually. They found the resulting system allowed public health workers to prioritize control strategies and target interventions to highest risk areas.

A number of researchers have developed applications for reporting or detecting mosquito breeding sites, as well as other information related to dengue outbreaks. Agrawal et al. [29] use a support vector machine and scale-invariant feature transform (SIFT) generated features to classify individual images as being breeding sites or not. Their approach relies on users to take photos of individual sites. On a test set of 78 images they achieved a binary classification accuracy of 82%. Mehra et al. [30] present a technique for classifying images into those containing puddles or not. They evaluate their technique on images taken with mobile phones, a hand-held thermal imaging camera, and retrieved using Google image search. Using an ensemble of naive Bayes classifiers and boosting they achieve a binary classification accuracy of 90% on images that have both RGB and thermal information. Quadri et al. [31] present TargetZika, a smartphone application for citizens to report breeding sites using photos and descriptions. They provide no automated classification of the photos but rather rely on users to label them from a menu. They use the data to produce risk maps but do not validate them. Mosquito Alert [32] is a similar smartphone application that allows users to report breeding sites and mosquitos with photos and descriptions. It uses crowdsourcing to identify photos. Reports are displayed on a map on the Mosquito Alert website. All of these previous approaches either require manual effort to first locate possible breeding sites in images or require users or the crowd to manually identify them. In contrast, the approach presented in this paper performs both object localization and classification and can be used on a wide variety of geotagged images taken from a horizontal perspective.

Some researchers have manually extracted features from GSV data for environmental monitoring purposes. Rundle et al. [33] manually extracted features from street view data to audit neighborhood environments and compared the results to field audits. They found a high level of concordance for features that are not temporally variable. Rousselet et al. [34] manually extracted species occurrence data for the pine processionary moth from GSV images and compared the results to field data. The two were found to be highly similar.

Runge et al. [35] made use of the scene recognition convolutional neural net of Zhou et al. [36] to label GSV images and assembled them into maps to find scenic routes for autonomous vehicle navigation. Although their application differs from ours, their pipeline and the structure of their feature maps are similar to those in this study. Since we are interested in obtaining counts of numbers of breeding sites in a given region, in this study we make use of object detection networks. Recently, region proposal methods have yielded the highest performance in object detection [37]. Region proposal methods employ a mechanism that first iteratively segments the image and groups the adjacent segments based on similarity to hypothesize regions that may contain objects of interest and then use CNNs to identify objects in those regions. Girshick [38] introduced Fast Region-based Convolutional Neural Networks (Fast R-CNN) which reduced the running time of the detection network, making the region proposal computation the bottleneck. Recently, Ren et al. [39] introduced Faster R-CNN, which greatly improves the computational efficiency. By sharing convolutional features between the region proposal and detection networks, they reduce the computational cost of region proposal to near zero and achieve a frame rate of 5 frames per second on a GPU. Because of its accuracy and computational efficiency, Faster R-CNN is the technique used in the current study.

## Methods

We describe details of the three main components of our pipeline to detect and map containers in geotagged images, namely image retrieval, container detection, and data visualization.

### Image retrieval

The region from which to retrieve images is defined using a GeoJSON file. The first step is to generate points within the region from which to retrieve the GSV images. This is done by obtaining the polyline of each road from the Openstreetmap Overpass API [40] and then plotting points along each road at 50 meter increments. A distance of 50 meters gives complete image coverage without overlap.

With the points defined, images are downloaded using the GSV API [41]. Since the API does not support retrieving the entire 360-degree scene as one image, five images are retrieved 72 degrees apart and at a field of view (FOV) of 75 and a pitch of -15 degrees. Each image has resolution 640 × 500 pixels. In addition, the metadata for each image is retrieved, consisting of the geo-coordinate and the year and month the image was taken. The Mapbox API is free of charge if the number of dynamic maps the Javascript API calls is less than 50,000 per month [42]. As of 2018, GSV images cost a maximum of 7 USD per 1000 panoramic images, depending on the monthly volume [43].

### Container detection

**Fig 1:**
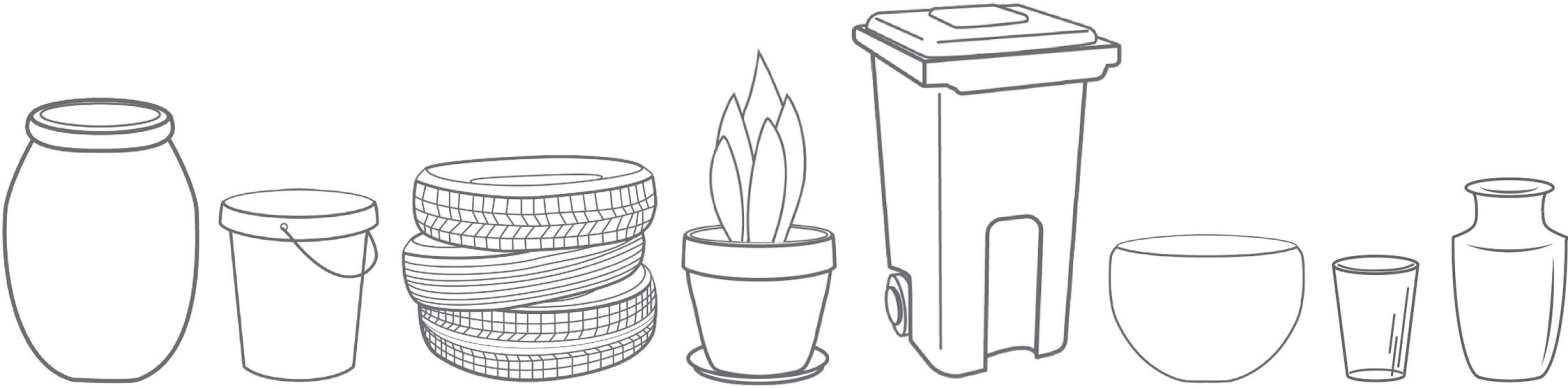
Common outdoor dengue vector breeding sites in Thailand (from left to right): large jar, bucket, old tire, potted plant, bin, ceramic bowl, cup, vase.

Dengue vector breeding sites consist of open containers of varying size that can contain water. The frequency of occurrence and the suitability of containers as breeding sites varies, with ceramic containers generally more suitable than plastic containers. While the importance of particular types of containers as breeding sites varies from country to country and even among geographic regions in a country [44], analysis of the research literature [45–48] as well as publications of the Ministry of Public Health of Thailand [49,50] reveals six outdoor container types that are consistently important across regions in Thailand. These are large ceramic jars, buckets, old tires, potted plants, bins, and bowls, as shown in Fig 1. This list was confirmed through consultation with local entomologists from Mahidol University. In general, large ceramic jars are the most important outdoor container type [45,50], being commonly used to store water near homes, particularly in rural areas.

Smaller containers such as bottles and cans are also possible breeding sites but are too small to detect in GSV images with high accuracy. Some areas such as construction sites, garbage dumps, and empty lots are commonly considered potential breeding sites [24,51] but GSV images do not provide sufficient coverage to detect containers in them. They may be best accounted for by using scene recognition techniques [36], like those used in the work of Runge et al. [35] and are not the focus of this study. In addition, indoor breeding sites and sites in backyards are not considered in this study due to the particular coverage of GSV images. Drone surveillance could potentially be used to detect containers in backyards and other outdoor areas not covered by GSV images.

Finding containers in GSV images falls into the class of problems known as object detection. We do this using the Faster R-CNN object recognition network of Ren et al. [39] which has state-of-the-art runtime performance. Object recognition networks employ region proposal algorithms to hypothesize object locations. Faster R-CNN combines a region proposal network (RPN) and object recognition network together by sharing the same common convolutional layer. At the convolution layer, the filters are trained to extract the appropriate features from the image, and convolution is computed by sliding the filters along the input image. The result is a two-dimensional matrix called a feature map. The RPN takes convolutional feature maps as inputs and predicts whether there is an object or not and also determines the bounding box of that object as the region proposal. Another fully connected neural network takes the regions proposed by the RPN and predicts object classes and creates bounding boxes surrounding the objects. To implement the Faster R-CNN network, we use TensorFlow which includes a number of architectural variations on Faster R-CNN that trade accuracy for speed and memory usage [52]. We use the architecture of Faster R-CNN with ResNet-101 (101 layers residual neural network) which has close to the highest accuracy on the Microsoft Common Objects in Context (COCO) object detection dataset [53] yet still excellent runtime performance. Performing object detection on the close to 1 million images for the province of Nakhon Si Thammarat in Thailand took 95 hours of processing time on a PC with a 3.6GHz i7-7700 processor, 32 GB RAM, and a 1080 Ti graphics card.

**Fig 2:**
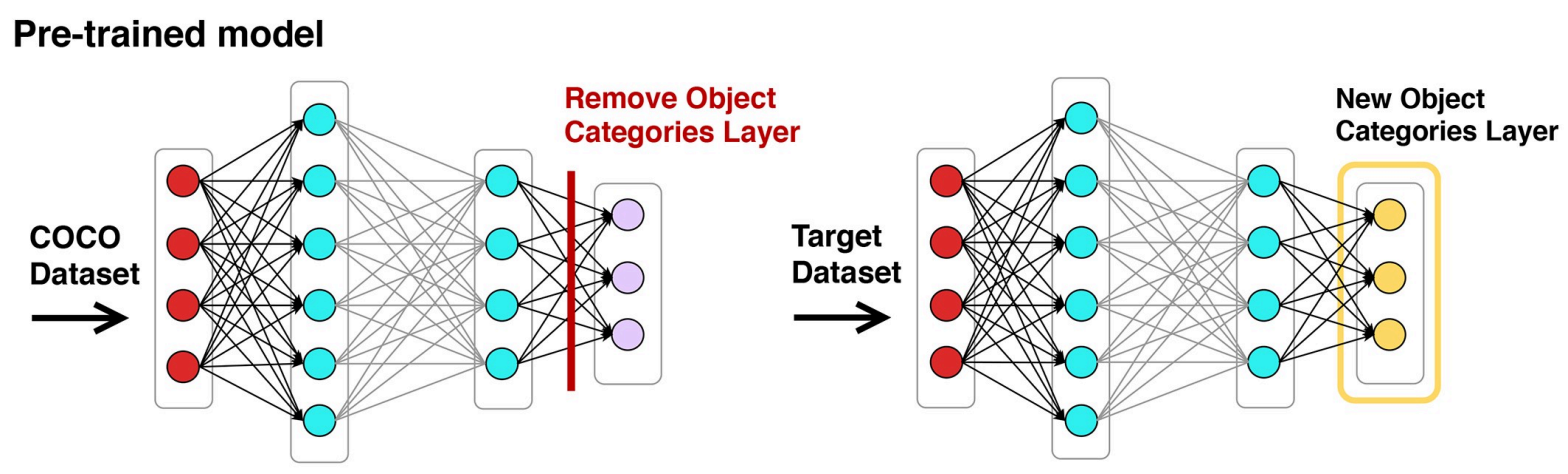
Performing transfer learning on a pre-trained model by replacing the output layer with new target classes.

Faster R-CNN includes the object categories bucket, potted plant, and bowl. In addition, the existing network categories for cup and vase work well for capturing short open and tall open containers, respectively. But the network does not include object categories for large jar, bin, and old tire. We thus used transfer learning to detect these categories [54]. Transfer learning leverages the features encoded in internal network nodes to enable learning of new categories with far fewer labeled examples than would normally be required. This is commonly done by stripping away the output layer of a pre-trained network, replacing it with the new categories to be learned, and then training the network on examples of those categories. In our case this was done by replacing the entire output layer of Faster R-CNN with our desired set of object categories, three of which were new and the remainder of which had been in the original Faster R-CNN, as shown in Fig 2. This network was then trained with the training data for all categories.

A training set of five thousand images was assembled from the COCO dataset [53], GSV images from Bangkok, and images gathered using Google image search on Thai language strings describing the container types. Data from COCO and Google image search was used to provide a sufficient number of images and data from GSV was used in order to provide images of the objects as they tend to appear in the particular context of the images to be processed. Table 1 shows the proportion of images and containers of each source in the training set.

**Table 1:** Number and percentage of images and containers from each source used for training and testing.

Source	Container type	Images	% images	Containers	% Containers
GSV images	All	2000	40.0	1901	18.4
Google image search	tire, jar, bin, bucket	1000	20.0	3755	36.3
COCO dataset	bowl, potted plant, cup, vase	2000	40.0	4689	45.3

Containers in the GSV images and those collected by Google image search were manually annotated by members of the research team with bounding boxes and container type labels by using the LabelImg [55] tool. Since each image can contain more than one container object, the collected images contained a total 10,345 containers: 2,318 old tires, 1,110 large jars, 1,385 buckets, 2,758 potted plants, 135 bins, 947 bowls, 930 cups, and 762 vases. Distinguishing a discarded old tire from a tire attached to a vehicle is difficult, so we solved this problem by adding vehicle as an object category and eliminating tires that have bounding boxes that substantially overlap with the bounding box of a vehicle.

The dataset was randomly split into 90% of the images for training and 10% for testing. To avoid overfitting the model to the training data, we applied the standard approach of early stopping during training. Early stopping is a form of regularization used to avoid overfitting when training a learner with an iterative gradient descent method like in Faster R-CNN.

**Fig 3:**
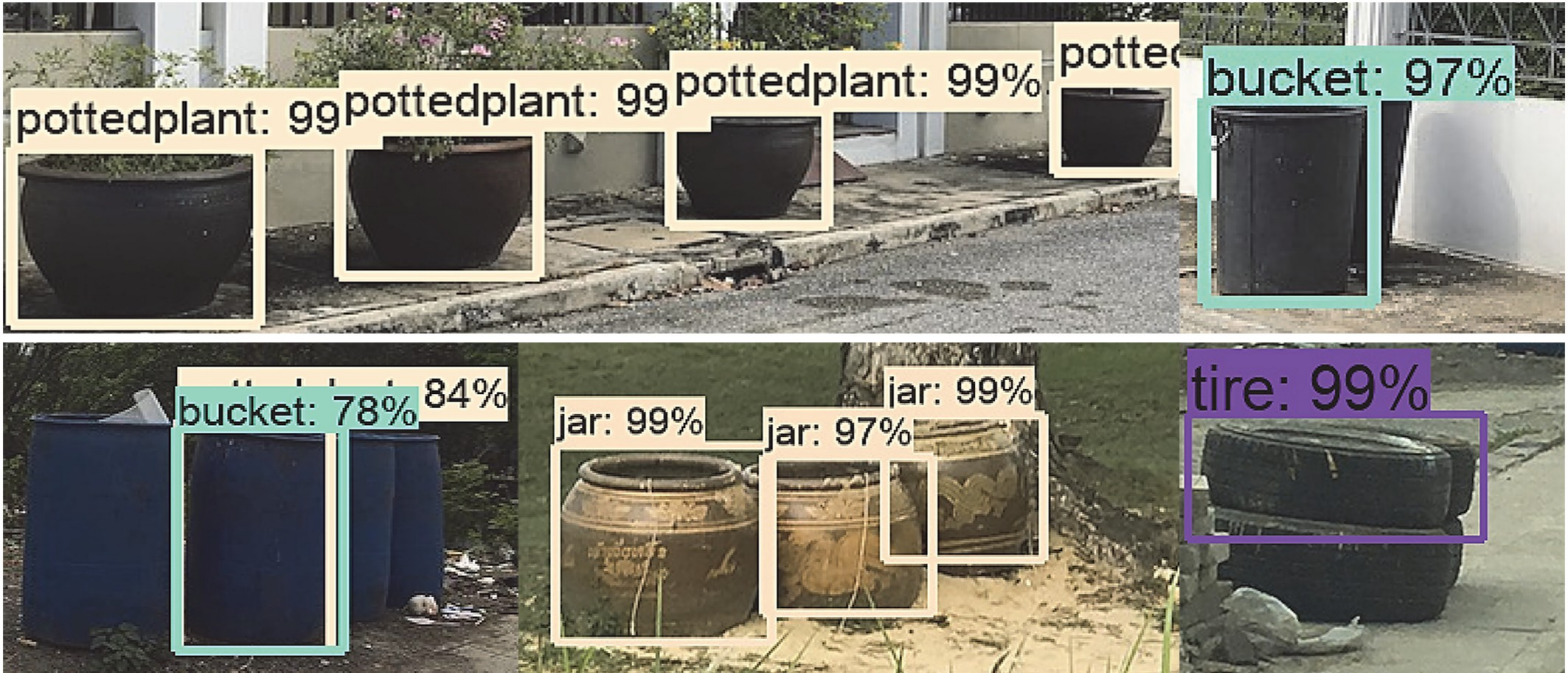
Examples of containers detected by using Faster R-CNN with new transferred categories.

Fig 3 shows examples of detected containers using the network resulting from transfer learning. The lower left image in Fig 3. illustrates a circumstance where the algorithm does not detect the containers correctly. The image contains four bins, but the algorithm is unable to detect some of the bins due to occlusion, poor lighting conditions, and low contrast with the background in the image. In addition, the algorithm incorrectly tagged one bin as a bucket and one as a potted plant, with the probabilities of 0.78 and 0.84, respectively. Detailed evaluation of the object detection accuracy is provided in the Result and Discussion section below.

### Data visualization dashboard

**Fig 4:**
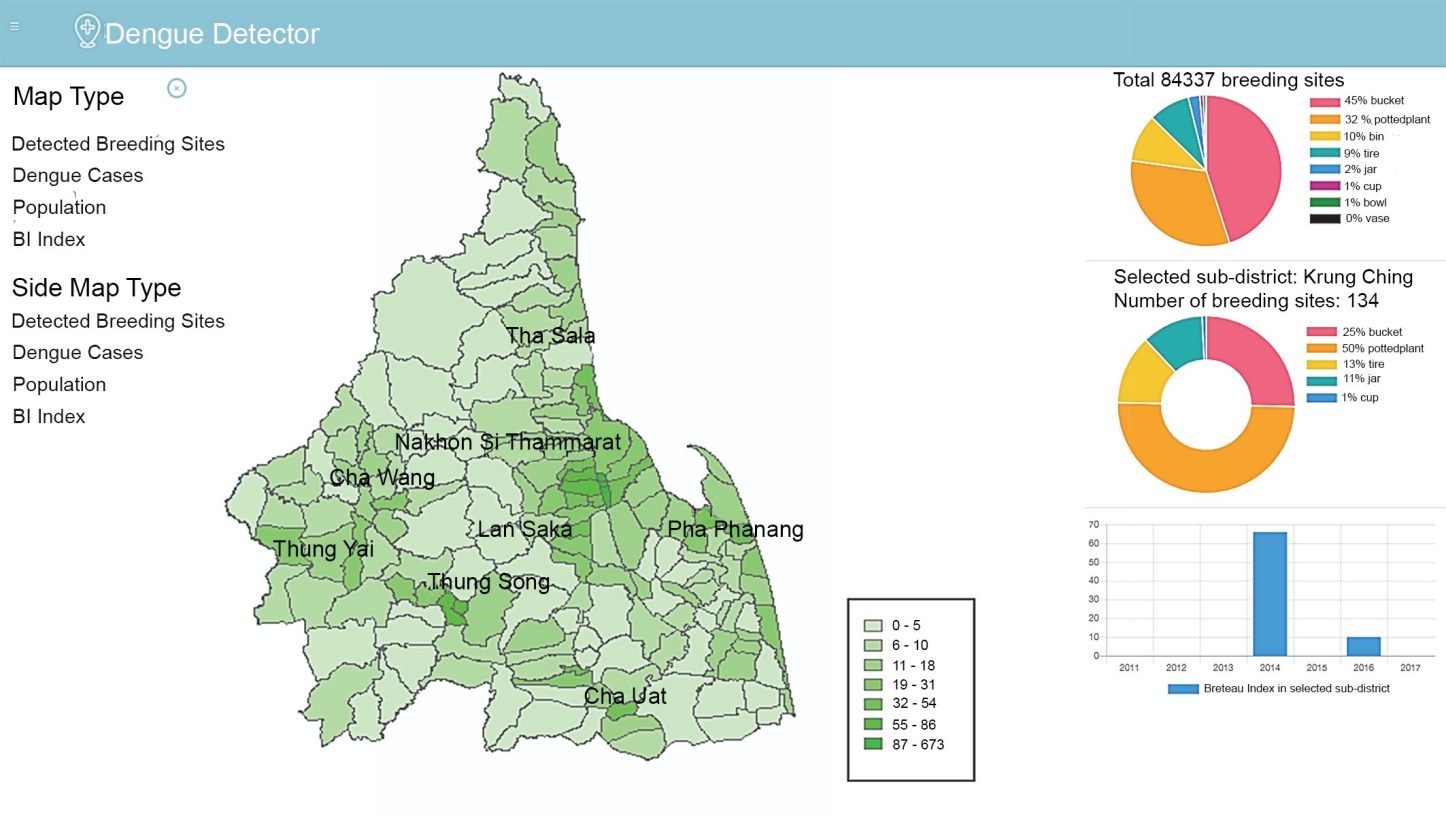
Information visualization dashboard. The choropleth map displays container densities for all sub-districts in Nakhon Si Thammarat province. The top chart on the right shows relative percentages of container types in the whole province. The second and third charts show statistics for the selected sub-district, in this case Krung Ching. When hovering over a subdistrict the data for the subdistrict is displayed. The choropleth map in this figure was produced using ArcGIS version 10.4 (Esri, Redlands, CA, USA). Source of shapefile: United Nations Office for the Coordination of Humanitarian Affairs https://data.humdata.org/dataset/thailand-administrative-boundaries.

The dashboard, shown in Fig 4, provides visualization of various data relevant to dengue risk, including container density, dengue incidence, Breteau index, population demographics, rainfall, and temperature. The data is displayed in terms of choropleth maps and graphs using Mapbox JS [41]. The maps are created by using a GeoJSON file as input and then applying a data-driven styling approach which allows the visualization of polygons on the map with varying colors based on the data [41]. Three charts are visible on the right side of the dashboard. The first chart displays statistics for the entire province while the other two charts display statistics for the selected sub-district. Users can filter the data to display only a certain year or season. Similarly, users can filter containers to display data for only certain types of containers. Each map has an additional mouse hover overlay where the exact value of the variable is shown.

## Results

In this section, we evaluate the accuracy of the object recognition technique in detecting containers in GSV images. We then present detailed statistics on container counts over three provinces in Thailand: Bangkok, Krabi, and Nakhon Si Thammarat. We compare container counts from GSV images to available manual counts. Finally, we evaluate the correspondence between container density values and Breteau index values from manual surveys in Nakhon Si Thammarat by computing correlations and creating multi-variate linear regression models.

Krabi province was chosen because it consistently has one of the highest dengue incidences in Thailand. Nakhon Si Thammarat was chosen because it has the greatest availability of manual larval survey data. Bangkok was chosen because, as the most urbanized and highly populated area of Thailand, it provides a contrasting environment to the other two provinces.

### Evaluation of object recognition

We use two metrics to evaluate container detection: (1) detection of containers, grouping all eight types together, and (2) detection along with categorization into one of the eight types. For the measurement of object recognition accuracy, we use the standard approach of determining the agreement between each detection bounding box with ground truth boxes in an image by calculating area of intersection over union (IoU). An IoU value of 0.5 or greater is considered to be a true positive [56]. An undetected object is counted as a false negative and a falsely detected object is counted as a false positive. Table 2 shows the performance on the test set which was a randomly selected 10% of the entire dataset of five thousands images described above. Accuracy is shown in terms of precision, recall and F1 score. Precision is defined as the ratio of correctly predicted positive containers to the total predicted positive containers from the images. Recall is defined as the ratio of correctly predicted positive containers to the total containers in the images. F1-score is the weighted average of precision and recall. The results for container detection are shown in the last column: precision is 0.90, recall is 0.92, and the F-score is 0.91. Results for the detection along with classification are shown in the remaining columns.

**Table 2:** Object recognition accuracy at 0.5 recognition confidence threshold for each category of container and grouping all container types. The average precision is calculated from the precision/recall curve by taking the average over all recall levels.

	Bin	Ceramic Bowl	Bucket	Large Jar	Cup	Potted Plant	Old Tire	Vase	All container types
**True object counts**	43	101	168	130	86	288	183	63	1062
**Precision**	1.00	0.78	0.83	0.94	0.76	0.89	0.92	0.79	0.90
**Recall**	0.23	0.89	0.94	0.82	0.91	0.94	0.93	0.86	0.92
**F-score**	0.37	0.83	0.88	0.88	0.83	0.91	0.92	0.82	0.91
**Average Precision**	0.42	0.51	0.86	0.71	0.46	0.75	0.81	0.63	-

The highest F-scores are achieved for potted plant (0.91) and old tire (0.92). The bin category has a high precision but low recall presumably because bins and buckets are very similar in shape so that some bins are wrongly tagged as buckets; this also lowers the precision of the bucket category. Note also that there is typically a tradeoff between precision and recall, so the perfect precision of the bin category is obtained at the cost of low recall.

### Analysis of container counts

**Fig 5:**
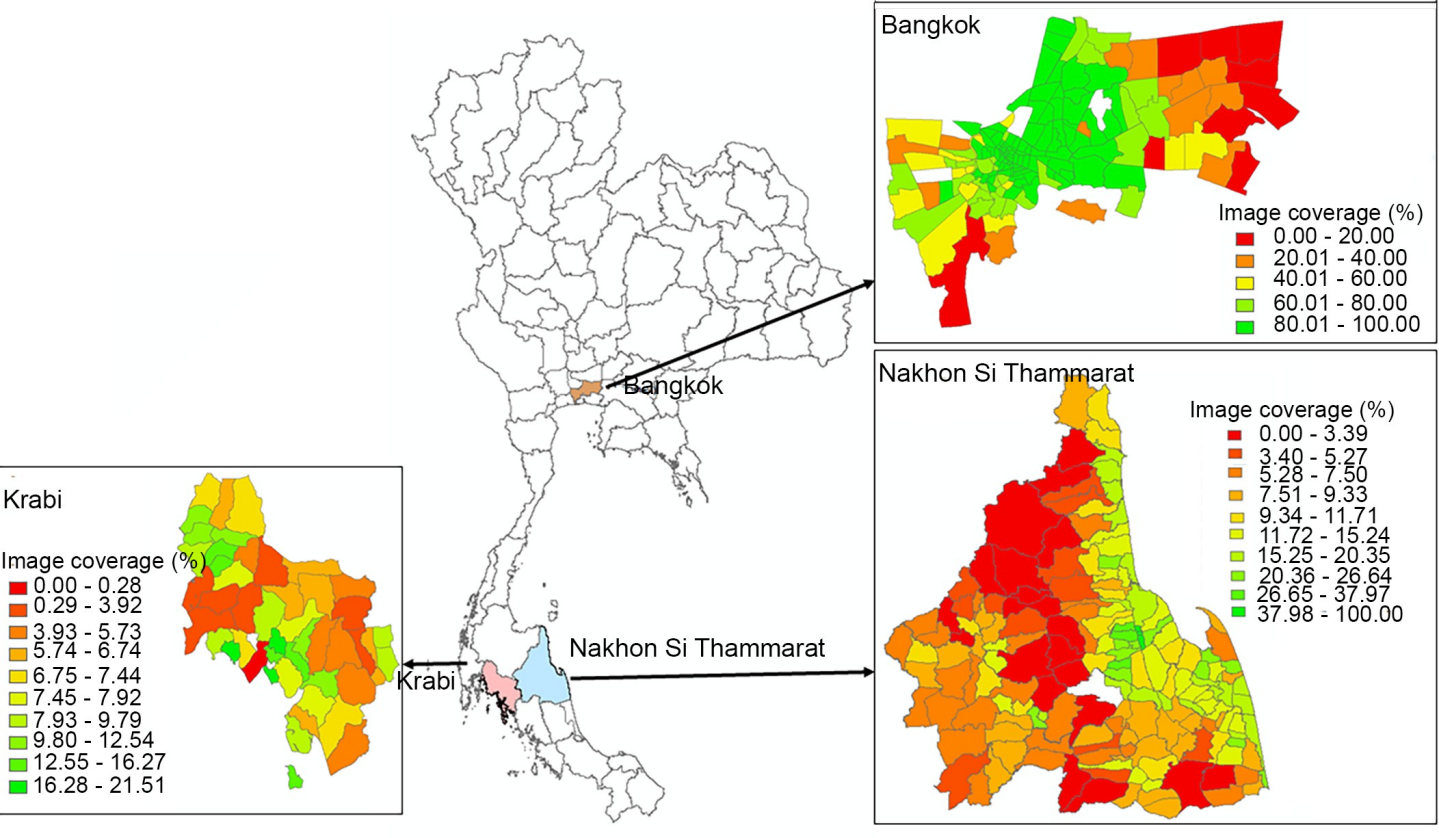
Image coverage in each province. Choropleth map produced using ArcGIS version 10.4 (Esri, Redlands, CA, USA). Source of shapefile: United Nations Office for the Coordination of Humanitarian Affairs https://data.humdata.org/dataset/thailand-administrative-boundaries). Note: White color means no image coverage.

Our software was used to retrieve GSV images from Bangkok (790,450 images), Nakhon Si Thammarat (958,027 images) and Krabi provinces (386,819 images) at every 50 meters and to detect all containers in those images. Details are shown in Tables A—C in the S1 Text. Percentage image coverage of the three provinces varied considerably. Bangkok had the best image coverage at a mean of 77.06% of total area over all districts, followed by Nakhon Si Thammarat at 8.40%, and Krabi at 7.31%. Although Bangkok has a smaller number of images than Nakhon Si Thammarat, the image coverage is by far the highest because the land area is much smaller. Fig 5 shows choropleth maps of percentage image coverage at the district level for the three provinces. Coverage tends to be highest in the main population centers and lower in more rural areas. This can be seen clearly in the map of Bangkok, where image coverage is highest in the central area. Percentage image coverage also varied considerably over the districts within each province. Bangkok had 100% image coverage for 21 out of 49 districts and a low of 15.45% for one district. In Nakhon Si Thammarat the coverage ranged from 19.7% to 2.4% and in Krabi from 11.36% to 5.15%.

**Fig 6:**
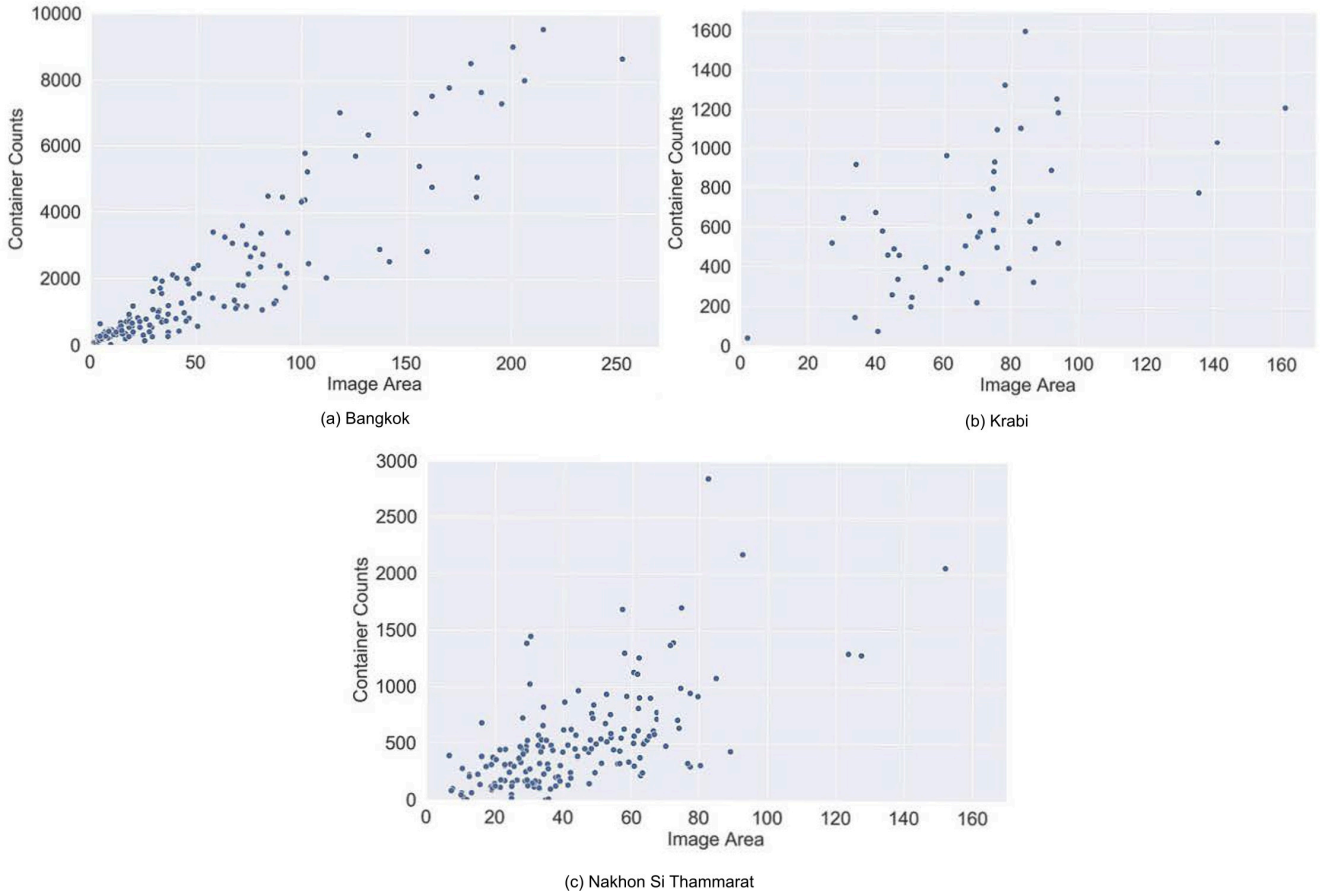
Container counts vs area covered by GSV images (km^2^) in a) Bangkok, b) Krabi, and c) Nakhon Si Thammarat.

A total of 298,391 containers were identified in Bangkok, 84,609 in Nakhon Si Thammarat, and 30,025 in Krabi. These counts lie in stark contrast to the number of images available for each province, with Nakhon Si Thammarat having 21% more images than Bangkok but 72% fewer containers. But within each province there is a fairly strong relationship between container count and the area covered by GSV images, as illustrated by Fig 6, which shows scatter plots of container counts vs image coverage in km^2^ in each province at the sub-district level. The Pearson correlations between container count and image coverage are 0.916 (p-value 0.000) for Bangkok, 0.558 (p-value 0.000) for Krabi and 0.673 (p-value 0.000) for Nakhon Si Thammarat.

Next we examined container density. Due to the limited availability of accurate shapefiles for Bangkok, we were not able to gather GSV images for Phra Khanong district and for nine sub-districts in other districts. These were left out of the calculations of density values so as not to bias the values down. Container density varied considerably. Bangkok had the highest container density (containers/km^2^ image area) over districts (Mean = 358.90, Standard variation (SD) = 119.79), followed by Nakhon Si Thammarat (Mean = 98.71, SD = 32.56), and then Krabi (Mean = 84.76, SD = 24.87). The highest container density of 729.75 was found in Din Daeng district of Bangkok. Container density per population was markedly more uniform across the three provinces but showed considerable variation among districts within the provinces.

**Fig 7:**
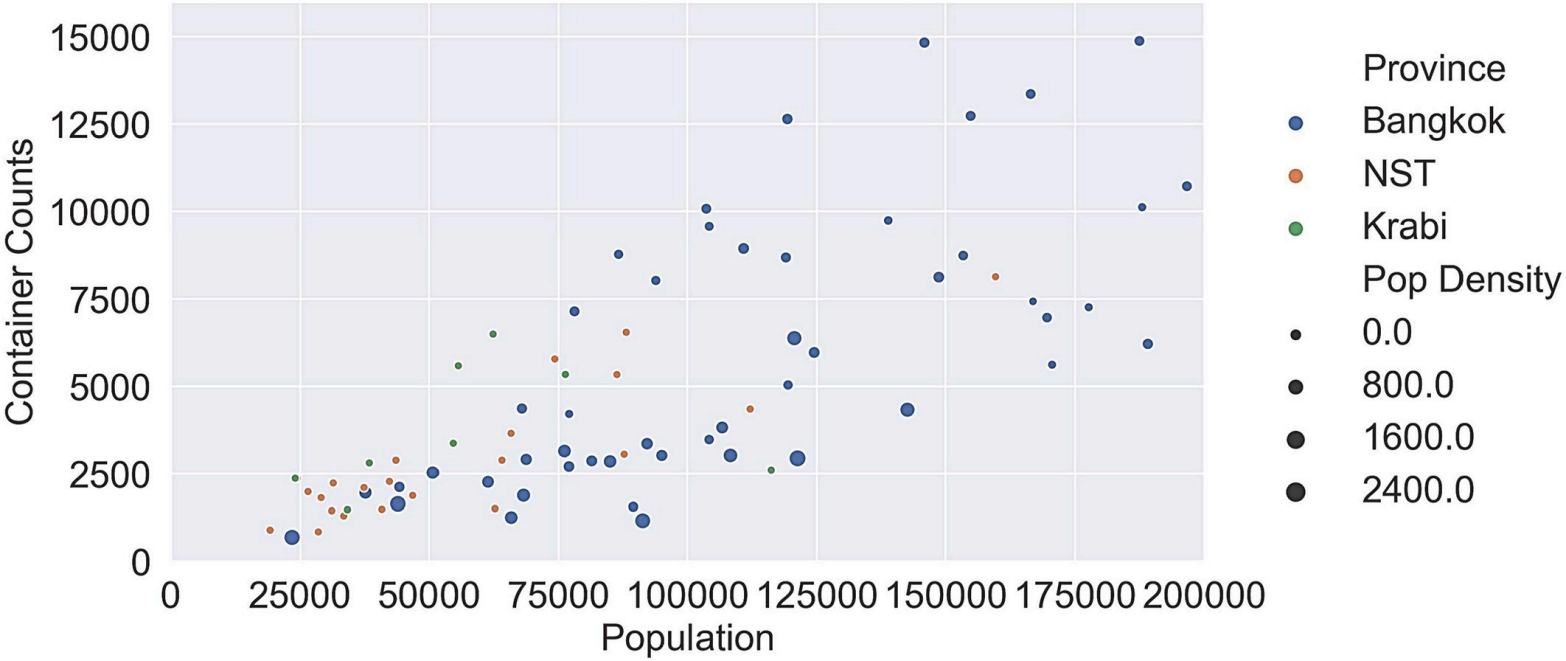
Container count vs population at the district level, color coded by province. Each datum is sized according to population density. (Note: NST = Nakhon Si Thammarat, Pop Density = population density).

Krabi had the highest container density by population (Mean = 7.12, SD = 2.90), followed by Bangkok (Mean = 5.30, SD = 3.19) and Nakhon Si Thammarat (Mean = 5.20, SD = 1.64). The highest density by population was found in Khanna Yao district of Bangkok at 17.71 containers per 100 population. Fig 7 shows a bubble chart of container counts vs population for all three provinces at the district level. Bubble size indicates population density. Mueang Nakhon Si Thammarat district from Nakhon Si Thammarat with population = 267,984, container counts = 19,915, population density = 52.737 is an outlier and was excluded from the plot. It can be seen that container counts tend to increase with population. The number of containers is well correlated with population in Nakhon Si Thammarat (Pearson correlation = 0.804, p<0.001) and moderately in Bangkok (Pearson correlation = 0.654, p = <0.001. For Krabi there are too few districts to compute a meaningful correlation.

**Fig 8:**
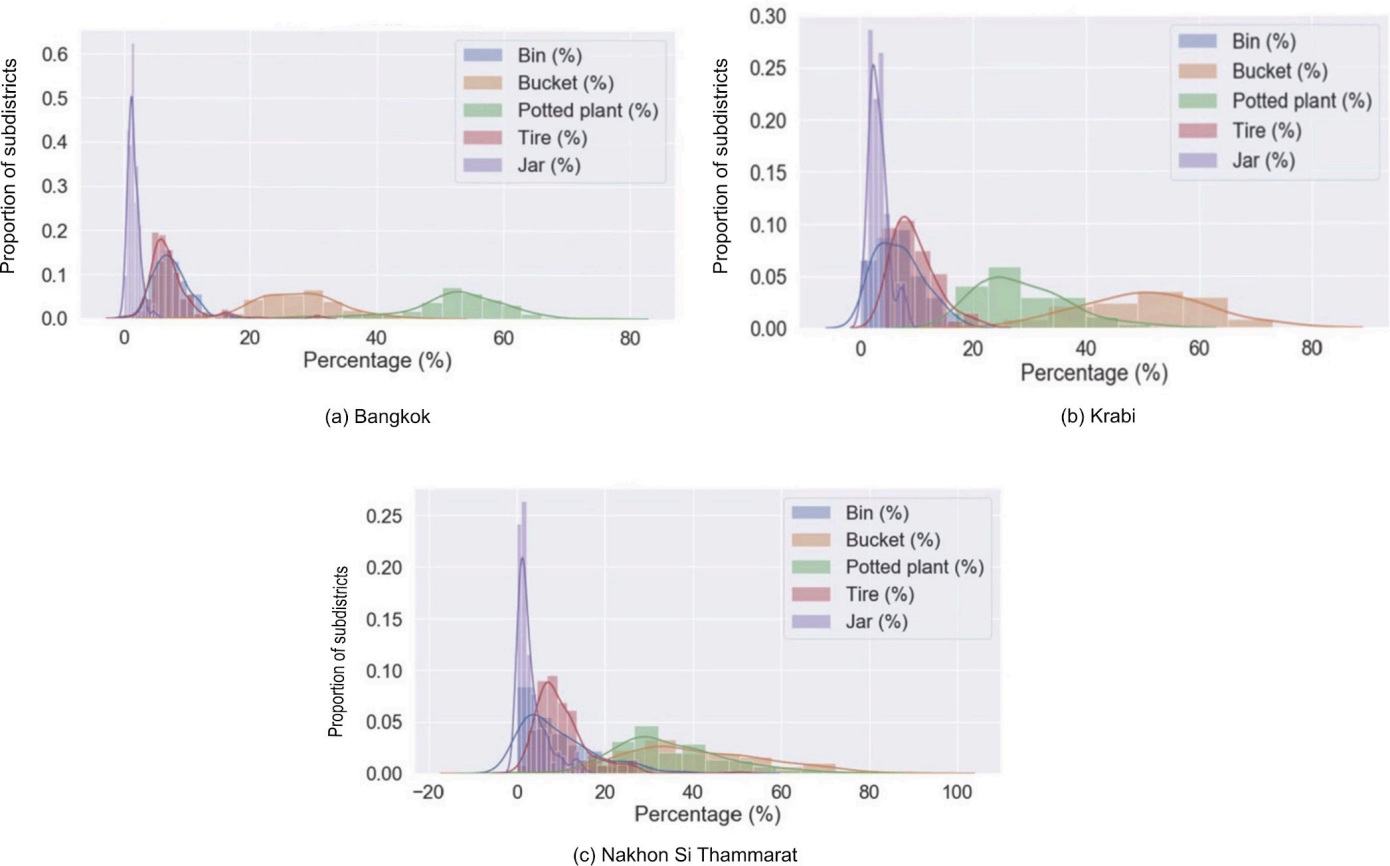
Distribution of relative prevalence of five most common container types (bin, bucket, jar, potted plant, tire) over sub-districts of (a) Bangkok, (b) Krabi and(c) Nakhon Si Thammarat provinces. Kernel density estimation was applied to smooth the values. (Note: Differences in bin widths are due to use of the Freedman-Diaconis rule for automatic binning used in plotting the distributions).

Among the eight detected categories of containers, potted plants and buckets account for the vast majority in all three provinces. In the highly urbanized area of Bangkok, buckets account for 29.96% of all containers, and potted plants for 51.84%. In the more rural provinces, the proportion is reversed. In Nakhon Si Thammarat, buckets and potted plants account for 45.14% and 32.08%, respectively and in Krabi they account for 52.27% and 27.56%, respectively. Fig 8 shows the variation of relative proportions of container types over all sub-districts of the three provinces. Bangkok has the least variation in prevalence of container types while Nakhon Si Thammarat has the highest.

**Fig 9:**
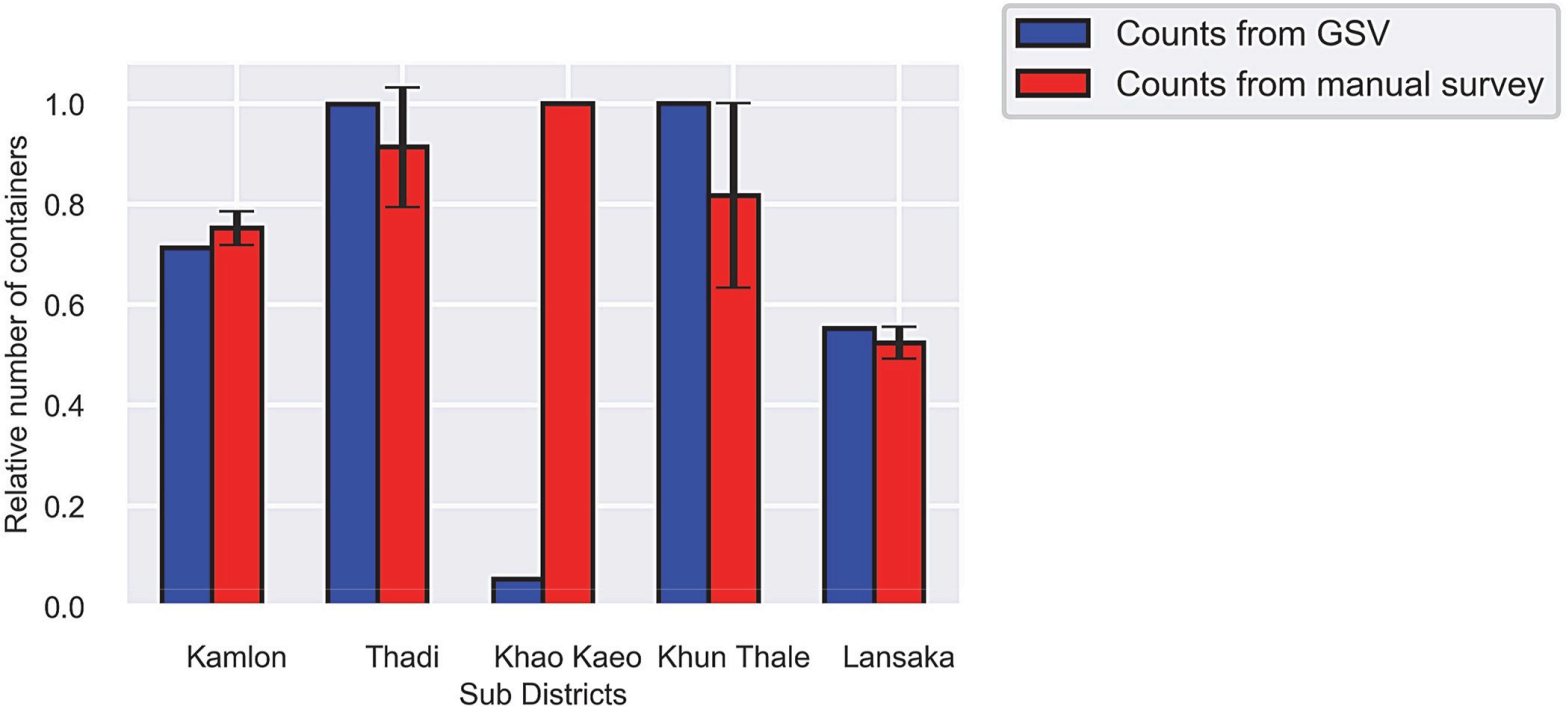
The relative numbers of containers in Lansaka District of Nakhon Si Thammarat from analysis of GSV images and from manual survey [57]. Values are shown relative to the highest count over the sub-districts for each study (95% confidence interval).

To validate the container counts from GSV images, we compared them with counts from available manual surveys. Chumsri et al. [57] conducted a study in five sub-districts of Lansaka district of Nakhon Si Thammarat in which they gathered indoor and outdoor container counts and larval counts in the wet and dry seasons of 2015. Our GSV images were taken during the dry season of 2016, so we compare our counts to their outdoor dry season counts. Since the absolute container counts from the two studies are not comparable due to different sampling techniques, we compare the relative counts over the five sub-districts in each study by normalizing by the highest count in each study. The result is shown in Fig 9. The relative counts over four of the sub-districts have strong agreement except for Khao Kaeo sub-district.

Table E in S1 Text shows the analysis of our container counts from GSV images over the five sub-districts. Khao Kaeo has the lowest coverage of GSV images at only 10.8 km^2^ and a container count of 24, compared to Khun Thale: 54.69 km^2^ with 446 containers, Kamlon: 24.49 km^2^ with 318 containers, Lansaka: 24.21 km^2^ with 246 containers, and Thadi: 23.10 km^2^ with 445 containers. Khao Kaeo also has the lowest percent image coverage of these sub-districts at 1.39%, which is the second lowest of all sub-districts in Nakhon Si Thammarat province. The low image area combined with the low percentage coverage could account for the large discrepancy between the container counts from GSV images and from the manual survey in Khao Kaeo.

**Fig 10:**
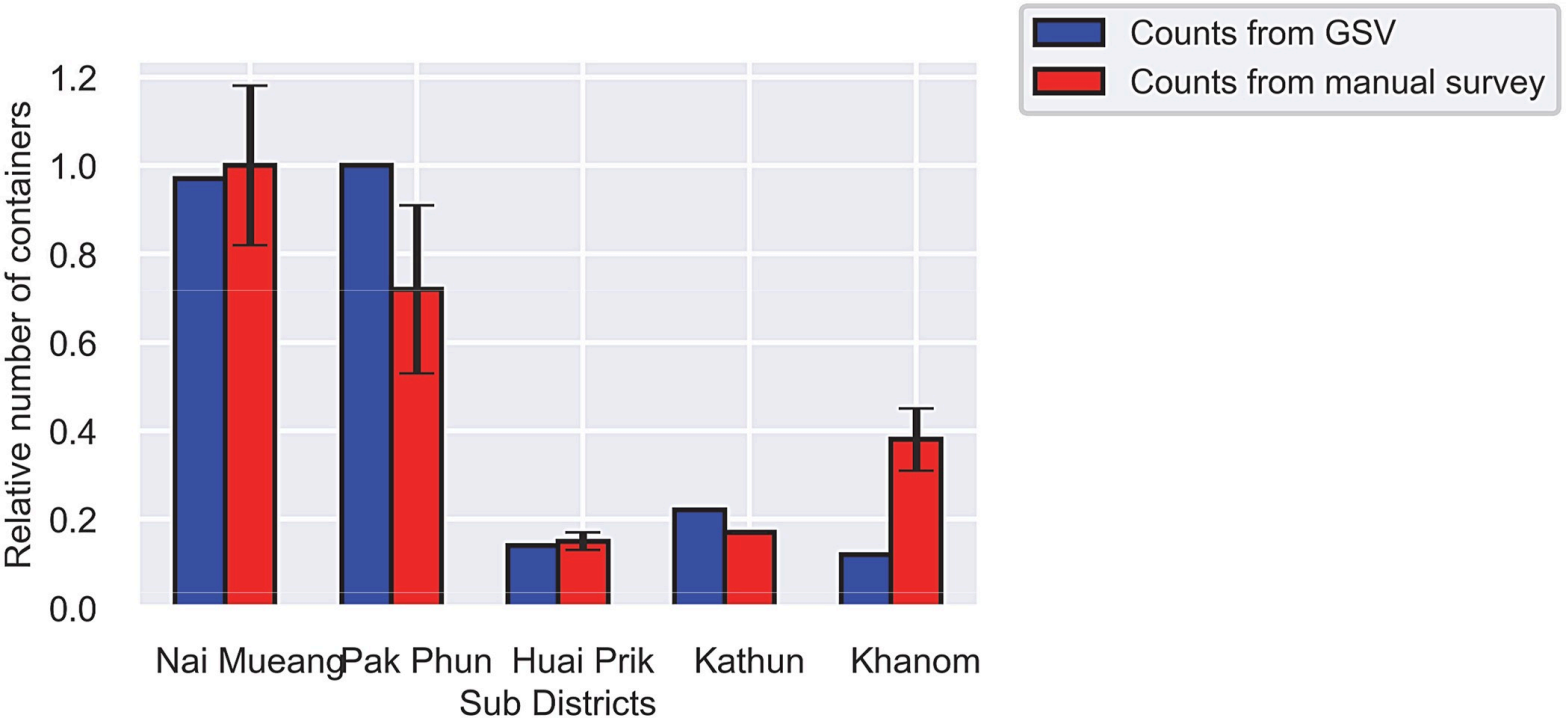
The relative numbers of containers in five sub-districts of Nakhon Si Thammarat from analysis of GSV images and from manual survey data of outdoor containers obtained from the Thai Ministry of Public Health. Values are shown relative to the highest count over the sub-districts for each study (95% confidence interval).

We additionally obtained manual container counts for sub-districts in Nakhon Si Thammarat from the Thai Ministry of Public Health [58]. Comparison of relative counts within this data is complicated by the fact that there was not a single survey sampling methodology consistently applied across sub-districts over time. We identified five sub-districts with outdoor container survey results from 2017 where the surveys inspected both villages and schools. We again compared relative container counts from the manual surveys with counts from GSV images, as shown in Fig 10. Analysis of correlation between the manual and GSV container counts shows a Pearson correlation of 0.9106 (p = 0.031).

### Comparison with larval survey data

Dengue vector abundance is influenced by a complex interplay of numerous factors. Climatic factors such as temperature and rainfall are widely known to influence Aedes abundance [59–61] and some studies have even shown that duration of daylight and wind velocity may be influential [62,63]. Vector abundance is also influenced by numerous factors related to human behavior and impact on the environment. These include construction practices, land cultivation, sanitation, domestic water storage, and crowded living conditions [63,64]. Arunachalam et al. [65] carried out a study of the eco-bio-social determinants of dengue vector breeding focused on geographic areas in six large and middle-sized Asian cities. Factors found to be significantly correlated with dengue vector density included number of containers, population density, and people’s knowledge and awareness of dengue and vector control activities. It was also found that public spaces contributed less to pupal production than domestic and peridomestic spaces. Across all study sites, unused and unprotected outdoor containers in shaded areas were found to be the highest contributor to pupal production. The importance of containers is underlined by the WHO Guidelines for Dengue Surveillance and Mosquito Control [66] which state that container management to reduce the sources of breeding habitats is one of the best approaches to controlling the dengue vector.

We evaluated the relationship between container counts determined from GSV images and dengue vector abundance by comparing container density values (containers/km^2^ land area) derived from GSV images with data from manual larval surveys at the village level. The computed container density values represent containers that contain or could contain water. We carried out the comparison for the province of Nakhon Si Thammarat, which was chosen because, among provinces in Thailand, it has the highest number of manual surveys in recent years and is consistently one of the provinces with the highest incidence of dengue cases. Container density values were generated by retrieving 958,027 GSV images from Nakhon Si Thammarat province and running them through the convolutional neural net for object recognition. Analysis of the metadata showed that the vast majority of images were taken in 2016. The first row of Table 3 shows the number of containers of each type over the 65 sub-districts. Detailed statistics are provided in Table D in the S1 Text.

**Table 3:** Description of detected containers used in comparison with larval surveys for entire year, dengue season and non-dengue season.

	N Subdistricts	Bin	Bowl	Bucket	Jar	Potted Plant	Tire	Cup	Vase	Total
Entire Year	60	3,171	160	15,513	631	8,946	3,024	22	221	31,688
Dengue Season	31	1,611	91	8,787	357	4,557	1,604	11	138	17,156
Non Dengue Season	48	2,364	125	12,789	551	7,263	2,414	18	177	25,701

We obtained seven years (2011–2017) of village-level larval survey data for Nakhon Si Thammarat from the Ministry of Public Health of Thailand. The larvae were manually identified by the village health volunteers who walked door-to-door and checked whether larvae were present in containers within or around the houses surveyed. The data from each survey was reported using three indices: Container Index, House Index, and Breteau Index. We use the Breteau Index (BI) for comparison since it is conceptually closest among these to our measure of container density and is considered the most useful of the three indices in estimating the *Aedes* density at a location [67]. So, the comparison we are making is between the number of positive containers per 100 houses inspected (including indoor and outdoor containers) and the number of outdoor containers that contain or could contain water.

To be meaningful, comparison of container density values and BI values should be done with data collected at roughly the same time. To maximize the amount of manual survey data, we used BI data from a 3-year time window: 2015–2017. This is justified by the assumption that while the location or presence of individual containers may change over time, the total number (absent major intervention efforts) is quite stable.

A complicating factor in our analysis is that the larval surveys were carried out at the village level. Producing corresponding container density values would require reliable village shapefiles, which are not available in Thailand. Since shapefiles are available for sub-districts, we carried out the comparative analysis at the sub-district level. As shown in Table 4, the BI for each sub-district was approximated by taking the average of the BI values of all villages in that sub-district. We excluded outliers from container density values and BI values by using three sigma (mean ± 3 SD) cutoff. This resulted in elimination of three data points for data over the entire year, one point for data over the dengue season, and four points for data over the non-dengue season, all at the upper end of the distribution. In addition, we eliminated data points for which the average BI in the sub-district had very high standard deviation. This resulted in the elimination of an additional two points for the entire year, one for the dengue season, and five for the non-dengue season. This left a total of 60 data points for the entire year (Table 4), 31 for the dengue season (Table 5), and 48 for the non-dengue season (Table 6).

**Table 4:** Description of Breteau Index data for the entire year used in analyses: Number of surveys per sub-district (N), mean value of BI, and SD.

	District	Sub-district	N	Mean	SD		District	Sub-district	N	Mean	SD		District	Sub-district	N	Mean	SD
1	Mueng	Tha Rai	1	68.0		21	Cha-uat	Khon Hat	1	37.5		41	Thung Yai	Kurae	24	17.2	3.7
2	Mueng	Kamphaeng Sao	1	57.5		22	Cha-uat	Ko Khan	7	17.9	6.2	42	Thung Yai	Prik	14	16.6	2.9
3	Mueng	Chai Montri	1	53.3		23	Cha-uat	Khuan Nong Hong	16	29.5	6.6	43	Thung Yai	Krung Yan	59	13.2	2.6
4	Mueng	Mamuang Song Ton	1	57.5		24	Cha-uat	Khao Phra Thong	11	31.8	4.6	44	Sichon	Thung Prang	2	35.5	13.4
5	Phrom Khiri	Phrom Lok	1	30.0		25	Cha-uat	Nang Long	1	40.0		45	Sichon	Sao Phao	1	50.0	
6	Phrom Khiri	In Khiri	2	50.0	3.5	26	Tha Sala	Tha Sala	2	56.2	15.9	46	Khanom	Khanom	2	10.0	7.1
7	Phrom Khiri	Thon Hong	2	33.5	2.1	27	Tha Sala	Sa Kaeo	2	15.0	8.5	47	Hua Sai	Laem	1	12.5	
8	Lan Saka	Tha Di	1	30.0		28	Tha Sala	Thai Buri	1	33.3		48	Bang Khan	Bang Khan	34	6.2	1.5
9	Lan Saka	Kamlon	2	33.5	0.7	29	Thung Song	Nong Hong	1	22.5		49	Bang Khan	Ban Lamnao	23	6.3	1.3
10	Chawang	Na Wae	11	15.7	2.8	30	Thung Song	Khuan Krot	2	36.2	12.4	50	Bang Khan	Wang Hin	3	7.5	0.0
11	Chawang	Huai Prik	6	16.2	1.0	31	Thung Song	Khao Ro	3	54.5	16.1	51	Bang Khan	Ban Nikhom	13	5.6	1.1
12	Chawang	Na Khliang	2	15.0	3.5	32	Thung Song	Thi Wang	4	41.2	9.2	52	Tham Phannara	Tham Phannara	2	27	0
13	Phipun	Kathun	8	19.0	2.0	33	Thung Song	Namtok	3	25.8	2.9	53	Chulabhorn	Thung Pho	10	33.1	5.3
14	Phipun	Khao Phra	2	22.1	1.2	34	Thung Song	Tham Yai	1	40.0		54	Chulabhorn	Na Mo Bun	1	32.5	
15	Chian Yai	Thong Lamchiak	1	15.0		35	Thung Song	Na Pho	5	43.0	9.4	55	Phra Phrom	Na Phru	2	9.5	4.9
16	Chian Yai	Karaket	1	32.5		36	Thung Song	Khao Khao	7	32.1	10.4	56	Phra Phrom	Na San	1	42.5	
17	Cha-uat	Cha-Uat	1	42.5		37	Na Bon	Na Bon	1	22.5		57	Nopphitam	Nopphitam	3	38.3	18.8
18	Cha-uat	Tha Pracha	5	31.2	7.9	38	Na Bon	Kaeo Saen	3	62.8	29.0	58	Nopphitam	Krung Ching	1	10	
19	Cha-uat	Wang Ang	17	23.0	6.2	39	Thung Yai	Tha Yang	5	13.5	1.4	59	Chang Klang	Chang Klang	2	20.5	0.7
20	Cha-uat	Ban Tun	10	30.2	3.6	40	Thung Yai	Thung Sang	1	12.5		60	Chaloem Phra Kiet	Thang Phun	2	10	0
												**Average Survey**			**5.88**	** **	** **

**Table 5:** Description of Breteau Index data for the dengue season used in analyses: Number of surveys per sub-district (N), mean value of BI, and SD.

	District	Sub-district	N	Mean	SD		District	Sub-district	N	Mean	SD		District	Sub-district	N	Mean	SD
1	Mueng	Tha Rai				21	Cha-uat	Khon Hat	1	37.5		41	Thung Yai	Kurae	24	17.2	3.7
2	Mueng	Kamphaeng Sao	1	57.5		22	Cha-uat	Ko Khan	7	17.9	6.2	42	Thung Yai	Prik	14	16.6	2.9
3	Mueng	Chai Montri	1	53.3		23	Cha-uat	Khuan Nong Hong	16	29.5	6.6	43	Thung Yai	Krung Yan	59	13.2	2.6
4	Mueng	Mamuang Song Ton	1	57.5		24	Cha-uat	Khao Phra Thong	11	31.8	4.6	44	Sichon	Thung Prang			
5	Phrom Khiri	Phrom Lok				25	Cha-uat	Nang Long				45	Sichon	Sao Phao			
6	Phrom Khiri	In Khiri				26	Tha Sala	Tha Sala				46	Khanom	Khanom			
7	Phrom Khiri	Thon Hong				27	Tha Sala	Sa Kaeo				47	Hua Sai	Laem			
8	Lan Saka	Tha Di				28	Tha Sala	Thai Buri				48	Bang Khan	Bang Khan	34	6.2	1.5
9	Lan Saka	Kamlon				29	Thung Song	Nong Hong	1	22.5		49	Bang Khan	Ban Lamnao	23	6.3	1.3
10	Chawang	Na Wae	11	15.7	2.8	30	Thung Song	Khuan Krot	2	36.2	12.4	50	Bang Khan	Wang Hin	3	7.5	0.0
11	Chawang	Huai Prik	6	16.2	1.0	31	Thung Song	Khao Ro	3	54.5	16.1	51	Bang Khan	Ban Nikhom	13	5.6	1.1
12	Chawang	Na Khliang	2	15.0	3.5	32	Thung Song	Thi Wang	4	41.2	9.2	52	Tham Phannara	Tham Phannara		
13	Phipun	Kathun	8	19.0	2.0	33	Thung Song	Namtok				53	Chulabhorn	Thung Pho	10	33.1	5.3
14	Phipun	Khao Phra				34	Thung Song	Tham Yai				54	Chulabhorn	Na Mo Bun	1	32.5	
15	Chian Yai	Thong Lamchiak				35	Thung Song	Na Pho	5	43.0	9.4	55	Phra Phrom	Na Phru			
16	Chian Yai	Karaket				36	Thung Song	Khao Khao				56	Phra Phrom	Na San			
17	Cha-uat	Cha-Uat	1	42.5		37	Na Bon	Na Bon				57	Nopphitam	Nopphitam			
18	Cha-uat	Tha Pracha	5	31.2	7.9	38	Na Bon	Kaeo Saen				58	Nopphitam	Krung Ching			
19	Cha-uat	Wang Ang	17	23.0	6.2	39	Thung Yai	Tha Yang	5	13.5	1.4	59	Chang Klang	Chang Klang			
20	Cha-uat	Ban Tun	10	30.2	3.6	40	Thung Yai	Thung Sang	1	12.5		60	Chaloem Phra Kiet	Thang Phun			
												**Average Survey**			**9.68**		

**Table 6:** Description of Breteau Index data for the non-dengue Season used in analyses: Number of surveys per sub-district (N), mean value of BI, and SD.

	District	Sub-district	N	Mean	SD		District	Sub-district	N	Mean	SD		District	Sub-district	N	Mean	SD
1	Mueng	Tha Rai	1	68.0		21	Cha-uat	Khon Hat				41	Thung Yai	Kurae	24	17.2	3.7
2	Mueng	Kamphaeng Sao				22	Cha-uat	Ko Khan				42	Thung Yai	Prik	14	16.6	2.9
3	Mueng	Chai Montri				23	Cha-uat	Khuan Nong Hong	16	29.5	6.6	43	Thung Yai	Krung Yan	59	13.2	2.6
4	Mueng	Mamuang Song Ton				24	Cha-uat	Khao Phra Thong	11	31.8	4.6	44	Sichon	Thung Prang	2	35.5	13.4
5	Phrom Khiri	Phrom Lok	1	30.0		25	Cha-uat	Nang Long	1	40.0		45	Sichon	Sao Phao	1	50.0	
6	Phrom Khiri	In Khiri	2	50.0	3.5	26	Tha Sala	Tha Sala	2	56.2	15.9	46	Khanom	Khanom	2	10.0	7.1
7	Phrom Khiri	Thon Hong	2	33.5	2.1	27	Tha Sala	Sa Kaeo				47	Hua Sai	Laem	1	12.5	
8	Lan Saka	Tha Di	1	30.0		28	Tha Sala	Thai Buri	1	33.3		48	Bang Khan	Bang Khan	34	6.2	1.5
9	Lan Saka	Kamlon	2	33.5	0.7	29	Thung Song	Nong Hong				49	Bang Khan	Ban Lamnao	23	6.3	1.3
10	Chawang	Na Wae	11	15.7	2.8	30	Thung Song	Khuan Krot	2	36.2	12.4	50	Bang Khan	Wang Hin	3	7.5	0.0
11	Chawang	Huai Prik	6	16.2	1.0	31	Thung Song	Khao Ro	3	54.5	16.1	51	Bang Khan	Ban Nikhom	13	5.6	1.1
12	Chawang	Na Khliang	2	15.0	3.5	32	Thung Song	Thi Wang	4	41.2	9.2	52	Tham Phannara	Tham Phannara	2	27.0	0.0
13	Phipun	Kathun	8	19.0	2.0	33	Thung Song	Namtok	3	25.8	2.9	53	Chulabhorn	Thung Pho	10	33.1	5.3
14	Phipun	Khao Phra	2	22.1	1.2	34	Thung Song	Tham Yai	1	40.0		54	Chulabhorn	Na Mo Bun			
15	Chian Yai	Thong Lamchiak	1	15.0		35	Thung Song	Na Pho				55	Phra Phrom	Na Phru	2	9.5	4.9
16	Chian Yai	Karaket	1	32.5		36	Thung Song	Khao Khao	7	32.1	10.4	56	Phra Phrom	Na San	1	42.5	
17	Cha-uat	Cha-Uat				37	Na Bon	Na Bon	1	22.5		57	Nopphitam	Nopphitam	3	38.3	18.8
18	Cha-uat	Tha Pracha	5	31.2	7.9	38	Na Bon	Kaeo Saen				58	Nopphitam	Krung Ching	1	10.0	
19	Cha-uat	Wang Ang	17	23.0	6.2	39	Thung Yai	Tha Yang	5	13.5	1.4	59	Chang Klang	Chang Klang	2	20.5	0.7
20	Cha-uat	Ban Tun	10	30.2	3.6	40	Thung Yai	Thung Sang				60	Chaloem Phra Kiet	Thang Phun	2	10.0	0.0
												**Average Survey**			**6.83**	** **	** **

**Fig 11:**
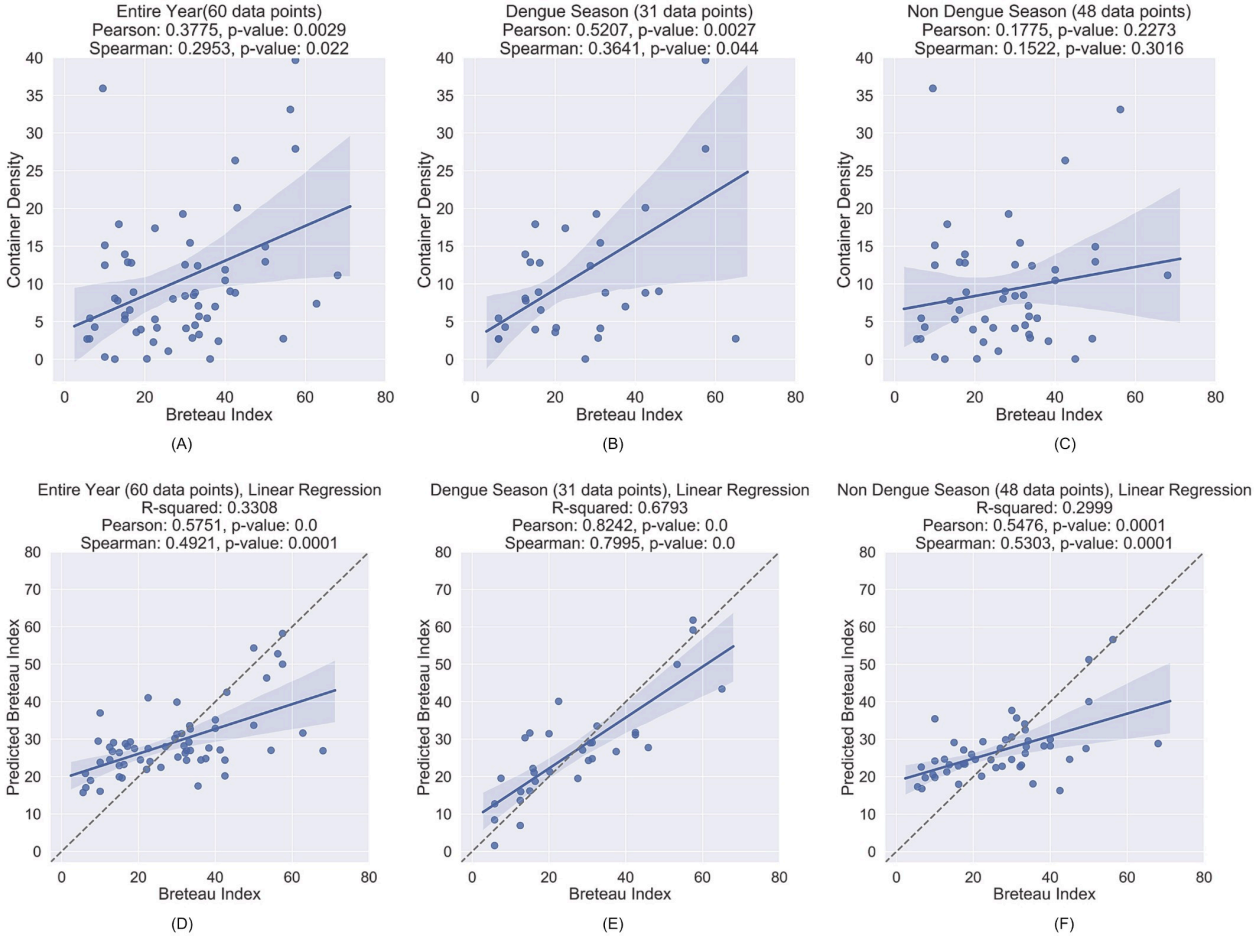
Correlation between container density by land area and BI for (A) entire year, (B) dengue season, and (C) non-dengue season, and predicted vs actual values of BI for multivariate linear regression model for (D) entire year, and (E) dengue season, and (F) non-dengue season. The solid line is a linear trendline which is an indication of the linear (Pearson) correlation between the two variables. (Note: shading shows the 99% confidence interval).

An initial straightforward approach to evaluating the agreement between container density and BI is to compute an overall container density by summing the numbers of containers of the eight different types. Computing the correlation between this and BI over 60 sub-districts for the entire year yields a Pearson correlation of 0.3775 (p = 0.0029) as shown in Fig 11A. This weak correlation is not surprising since we are measuring the relation between container density and BI during some months when there is little or no rain; thus few larvae in the counted containers. We would expect the correlation to naturally be low during the dry season and higher during the rainy season. To test this we separately measured the correlation with BI values collected during the wet dengue season, which in Nakhon Si Thammarat is June—November [68], and the remaining months, the non-dengue season.

For the dengue season, this left 31 sub-districts with BI data and for the non-dengue season, this left 48 sub-districts. Rows two and three in Table 3 show the numbers of containers of each type for the dengue and non-dengue seasons, respectively. Over the dengue season, the Pearson correlation is moderately strong 0.5207 (p = 0.0027), as shown in Fig 11B, while over the non-dengue season the Pearson correlation is a very weak 0.1775 (p = 0.2273), as shown in Fig 11C.

Vector abundance in a given area depends on container density as well as container productivity, with productivity often varying greatly among container types [57,65,69]. Thus, a more precise relation between container counts and BI can potentially be obtained by analyzing the relationship using the disaggregated counts of the various container types. We created multivariate linear regression models with container densities for the eight types of containers as the independent variables and BI as the dependent variable. Evaluation of the fitted linear model shows a moderately strong Pearson correlation with the BI values of 0.5751 (p < 0.0001) with R-squared of 0.3308 for entire year, a significantly high Pearson correlation of 0.8242 (p < 0.0001) with R-squared of 0.6793 for the dengue season, and 0.5476 (p = 0.0001) with R-squared of 0.2999 for the non-dengue season, as shown in Fig 11D, 11E and 11F, respectively. The standardized beta coefficients for the dengue season model, shown in Table 7, indicate that potted plants and large jars are the most important types of containers in predicting BI values within the 31 sub-districts. Interestingly, these are not the most prevalent types of containers in the sub-districts. The most prevalent are buckets (47.46%), potted plants (28.42%), and tires (10.53%). Large jars represent only 2.31% of the detected breeding sites. This result conforms to results from previous entomological studies of the dengue vector in Thailand which found that potted plants and large jars are two of the most important breeding site types. The Ministry of Public Health [49,50] reports that among larval surveys carried out throughout the country, 70.82% of *Aedes aegypti* larvae are found in large jars. In a study of *Aedes aegypti* breeding sites in Kamphaeng Phet, Thailand, Koenradt et al. [45] found earthenware jars to be responsible for 33.1% of pupae production. A study of dengue vector breeding sites in Nakhon Si Thammarat found that the number of positive containers was higher in earthen containers (e.g., potted plants and large jars) than in plastic ones [70]. This analysis demonstrates the value of our data driven approach in identifying important container types, which is recognized as being essential in effective dengue control [71].

**Table 7:** Absolute standardized coefficients and p-values from linear regression for dengue season. The largest absolute values are the most important variables in the regression model.

Absolute Standardized Coefficients								
	Potted plant	Large Jar	Bin	Cup	Bucket	Tire	Bowl	Vase
Beta	3.4828	2.0499	0.6684	0.4976	0.4501	0.3806	0.1897	0.0074
p-values	0.0000	0.0000	0.0570	0.0110	0.1400	0.1290	0.3960	0.9600

**Fig 12:**
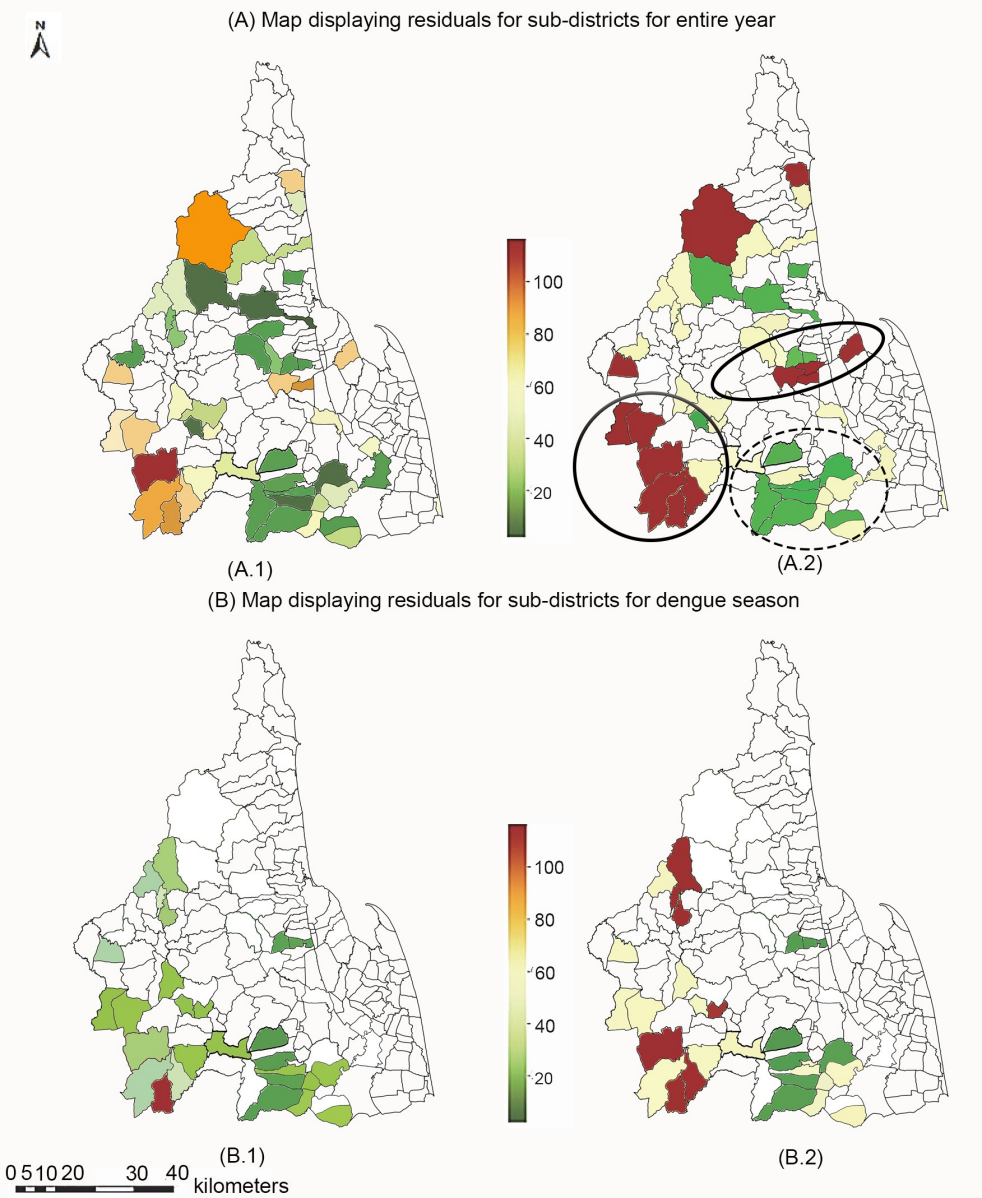
Choropleth maps of SAPE values for the multivariate linear models for (A) entire year, and (B) dengue season, where A.1, B.1 are gradient colormaps, and A.2, B.2 are thresholded colormaps using the 25% and 75% quantiles as threshold values. The dashed circle and solid circle delineate the clusters where the model fit are good and poor, respectively. White color represents subdistricts with no data. Choropleth map produced using ArcGIS version 10.4 (Esri, Redlands, CA, USA). Source of shapefile: United Nations Office for the Coordination of Humanitarian Affairs https://data.humdata.org/dataset/thailand-administrative-boundarieson) correlation between the two variables.

To understand conditions under which the linear regression models fit well and under which they do not, we carried out an analysis of the model residuals over the sub-districts using the symmetric mean absolute percentage error (SMAPE) which has the advantage of being independent of magnitude of the values being estimated. This was applied to the single value for each sub-district so that the value of n is just 1 and the formula becomes 2(|F—A|) / (|F| + |A|), where A is actual value and F is the predicted value; thus for clarity we use the term symmetric absolute percentage error (SAPE). Fig 12A.1 and 12A.2 show the SAPE values for the entire year using a gradient color scheme and thresholding, respectively. Fig 12B.1 and 12B.2 similarly show the SAPE values for only the dengue season using gradient color scheme and thresholding. Since the results are quite similar, we will restrict our discussion to the entire year, using the thresholded colormap which most clearly displays the areas where the models are accurate or inaccurate. The map uses 25% and 75% quantile threshold values to categorize sub-districts into three classes: good fit (dark green), average fit (yellow), and poor fit (dark red). In the figure we can observe some amount of clustering of regions of good fit and poor fit.

The solid circle delineates a cluster of six sub-districts where the model fit is poor. Four of the sub-districts are in Bang Khan district and the other two are in Thung Yai district, which are mountainous areas. A previous study by Preechaporn et al. [46] examining the effect of topography on key breeding sites in Nakhon Si Thammarat found that in these mountainous areas the key containers for *Aedes aegypti* were preserved areca jars and for *Aedes albopictus* were metal boxes. These two container types are not detected by our object recognition software.

The oval delineates another cluster of four sub-districts where model fit is poor. These sub-districts (Tha Rai, Mueang district; Khun Thale, Lan Saka district; Na Phru and Na San, Phra Phrom district) are urban areas with high population density. A plausible explanation is that in such urban areas, indoor containers represent a large proportion of breeding sites which cannot be detected in the GSV images. In urban environments, *Aedes aegypti* is more prominent than *Aedes albopictus* and the former prefer indoor breeding sites [72,73]. In a study of the effect of urbanization on the presence of *Aedes aegypti* and *Aedes albopictus* in Chiang Mai, Thailand, Tsuda et al. [74] found a larger number of mosquito larvae indoors than outdoors in their urban study area and the reverse in their rural study area.

The dashed circle in the figure delineates a cluster of sub-districts, mostly in Cha-Uat district, where the model fit is good. A previous study of the ecology of *Aedes* mosquitos in Kreang sub-district of Cha-Uat district [75] found plastic buckets to be the most common breeding sites. Our analyses show plastic buckets to be the most prevalent containers in Cha-Uat district (51.73%) as shown in Table B in S1 Text.

**Fig 13:**
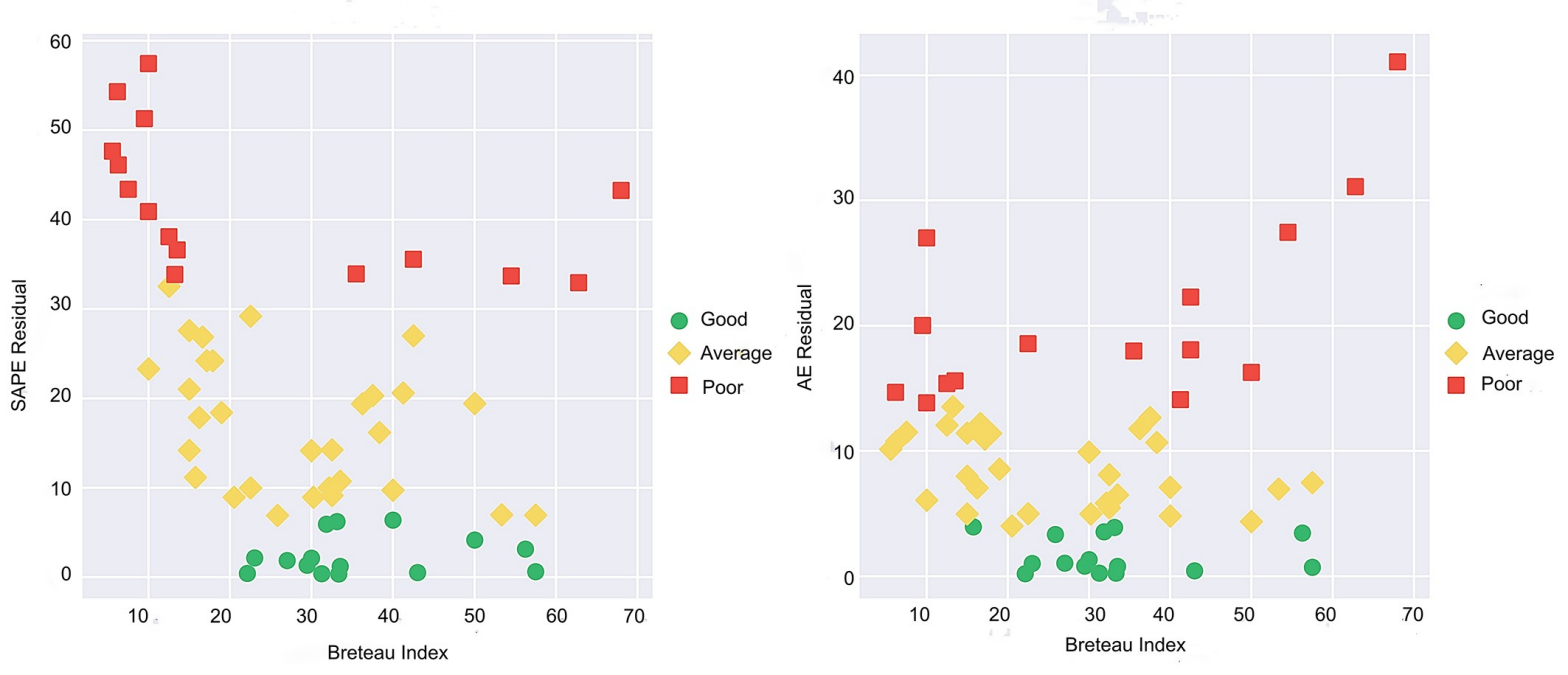
Scatter plots of (a) SAPE residual and (b) AE residual values of sub-district predictions versus Breteau index. The 25% and 75% quantiles are used as thresholds for the categorization into Good, Average, Poor.

Fig 13A and 13B show scatter plots of the SAPE and Absolute Error (AE) of the model predictions versus the BI values of the sub-districts. The AE is defined as the absolute value of the difference between the prediction and the actual value. The same thresholded color coding is used as in the map in Fig 12A.2. Accuracy tends to be good toward the middle range of BI values (between about 20 and 40) and is worse at low and high ends of the BI range. Two of these high BI value sub-districts, shown in red, correspond to two of the sub-districts with high population density discussed above.

## Discussion

We presented a pipeline to detect and map containers using images from Google Street View. The central component in this pipeline is the Faster R-CNN object recognition network from which we used five existing object categories in the network and used transfer learning to train an additional three. Evaluation on a test set of images yielded an F-score accuracy of 0.91 for the problem of detecting any of eight types of containers. While the eight object categories in the network cover a number of the most important container types for the dengue vector in Thailand, there are some notable missing types. Cement tanks are known to be important breeding sites throughout Thailand [47,48] but are not in Faster R-CNN and images to use for transfer learning are not readily available. Future work could collect a set of training images through crowdsourcing and/or by using the network of local healthcare volunteers of the Ministry of Public Health of Thailand. Based our experience with transfer learning of three object categories, we estimate that between a few hundred and a thousand images would be sufficient. In addition, numerous other container types are important breeding sites regionally. For example, one study in Nakhon Si Thammarat [46] found *Aedes aegypti* larvae mostly in preserved areca jars in mangrove and mountainous areas, and *Aedes albopictus* larvae mostly in preserved areca jars in mangrove areas and in metal boxes in mountainous areas. Such container types could also be added to produce a more comprehensive catalog of containers. Very small containers such as cans and bottles are difficult to recognize in GSV images. This could be partially addressed by using scene recognition techniques [36] to detect areas such as garbage dumps that have high concentrations of such containers.

Despite these limitations of container coverage, a simple multi-variate linear regression model relating densities of the eight container types with Breteau Index values for 31 sub-districts in Nakhon Si Thammarat province of Thailand yields an R-squared value of 0.6793 during the dengue season. In ongoing work, we are constructing risk models of dengue using rainfall, temperature, and population demographics, as well as the container densities from GSV images in order to understand and quantify the added value of this source of container density data in dengue risk mapping.

While GSV data is an excellent data source for evaluating the potential usefulness of the approach presented in this study, it has a number of limitations that make it less ideal for supporting practical control efforts. These limitations concern mostly temporal and spatial data coverage. As mentioned earlier, GSV data is updated only at infrequent intervals, with higher refresh rates in more urban areas and along larger roads. This limitation can be partially addressed through the use of existing crowdsourcing tools for gathering geotagged images, such as the smartphone applications Mapillary (www.mapillary.com) and Open Street Cam (openstreetcam.org). These applications allow anyone to easily create and share street view type images. In terms of the spatial coverage, GSV image coverage varies greatly, with coverage best in urban areas. Our analysis showed the image coverage of highly urbanized Bangkok to be 77.06% and the coverage of the more rural provinces of Nakhon Si Thammarat and Krabi to be 8.40% and 7.31%, respectively. Coverage also varied greatly among districts in the provinces. In addition, GSV images cover only areas along roads and so do not cover areas such as empty lots and back yards. For such areas, the use of drones offers a possible approach to gather fairly high-resolution images [76]. But use of drones has a number of challenges, including relatively high cost, specific training required to properly operate the drones, significant amount of time required to obtain images from large areas, sensitivity to local weather conditions, and regulations on flying over populated areas [77]. Because of the need to fly at an altitude to avoid obstacles, drones also typically provide images of lower resolution than street view images. Of course, none of the image-based techniques discussed here provide coverage of indoor containers. Since indoor containers can represent a significant portion of overall containers, particularly in urban areas, this is a fundamental limitation of image-based techniques. Despite these limitations, the results presented in this paper suggest that detection of containers in geo-tagged images may be a useful tool in creation of dengue risk maps.

The source code for the trained Faster R-CNN network and the container counts used in our study are available upon request.

## Supporting information

S1 Text Table A. Area, image coverage, population, and statistics of detected containers at the district level in Bangkok, Thailand Table B. Area, image coverage, population, and statistics of detected containers at the district level in Nakhon Si Thammarat, Thailand Table C. Area, image coverage, population, and statistics of detected containers at the district level in Krabi, Thailand Table D. Statistics of detected containers for sub-districts where BI values were collected during the dengue season. Table E. Statistics for detected containers in sub-districts of Lansaka district of Nakhon Si Thammarat.
(DOCX)
