# Supplementary material for: Large scale detailed mapping of dengue vector breeding sites using street view images
Source: PLoS Negl Trop Dis. 2019 Jul 29;13(7):e0007555. doi: 10.1371/journal.pntd.0007555 (PMC6687207; doi:10.1371/journal.pntd.0007555)
Supplement: S1 Text — Table A. Area, image coverage, population, and statistics of detected containers at the district level in Bangkok, Thailand Table B. Area, image coverage, population, and statistics of detected containers at the district level in Nakhon Si Thammarat, Thailand Table C. Area, image coverage, population, and statistics of detected containers at the district level in Krabi, Thailand Table D. Statistics of detected containers for sub-districts where BI values were collected during the dengue season. Table E. Statistics for detected containers in sub-districts of Lansaka district of Nakhon Si Thammarat. (DOCX) [file pntd.0007555.s001.docx]

**Table A.** Area, image coverage, population, and statistics of detected containers at the district level in Bangkok, Thailand

| District Name | Area (km^2^) | Area of GSV images (km^2^) | GSV image coverage (%) | Population | Number of containers | Containers per km^2^ land | Containers per km^2^ image area | | Containers per 100 persons |  | Relative proportions of container types (%) | | | | | | | | | | | | | | | |
| --- | --- | --- | --- | --- | --- | --- | --- | --- | --- | --- | --- | --- | --- | --- | --- | --- | --- | --- | --- | --- | --- | --- | --- | --- | --- | --- |
|  |  |  |  |  |  |  |  |  |  |  | Jar | | Bucket | | Potted plant | | Tire | | Bin | | Bowl | | Cup | | Vase | |
| Phra Nakhon | 5.53 | 5.53 | 100.00 | 52,522 | 2,527 | 456.96 | 456.96 | 4.81 |  | 0.87 | | 20.14 | | 61.06 | | 4.00 | | 6.61 | | 3.44 | | 2.89 | | 0.99 | |  |
| Dusit | 11.69 | 5.86 | 50.13 | 98,450 | 1,158 | 99.06 | 197.61 | 1.18 |  | 0.86 | | 25.22 | | 54.40 | | 6.65 | | 8.98 | | 2.07 | | 1.73 | | 0.09 | |  |
| Nong Chok | 248.15 | 38.33 | 15.45 | 167,844 | 7,432 | 29.95 | 193.90 | 4.43 |  | 2.27 | | 36.05 | | 40.76 | | 9.08 | | 10.71 | | 0.63 | | 0.40 | | 0.09 | |  |
| Bang Rak | 4.14 | 4.14 | 100.00 | 47,308 | 1,956 | 472.46 | 472.46 | 4.13 |  | 0.82 | | 24.23 | | 54.70 | | 7.31 | | 6.44 | | 3.48 | | 2.30 | | 0.72 | |  |
| Bang Khen | 42.28 | 39.62 | 93.71 | 190,828 | 14,879 | 351.92 | 375.54 | 7.80 |  | 1.76 | | 29.18 | | 51.51 | | 5.56 | | 10.36 | | 0.87 | | 0.57 | | 0.19 | |  |
| Bang Kapi | 28.47 | 28.47 | 100.00 | 148,392 | 14,832 | 520.97 | 520.97 | 10.00 |  | 1.21 | | 29.79 | | 54.15 | | 5.05 | | 7.69 | | 1.05 | | 0.84 | | 0.21 | |  |
| Pathum Wan | 8.29 | 8.29 | 100.00 | 49,594 | 2,118 | 255.49 | 255.49 | 4.27 |  | 0.85 | | 21.44 | | 56.19 | | 9.87 | | 6.09 | | 2.55 | | 2.27 | | 0.76 | |  |
| Pom Prap Sattruphai | 2.58 | 2.58 | 100.00 | 47,450 | 1,637 | 634.50 | 634.50 | 3.45 |  | 0.73 | | 22.97 | | 55.96 | | 10.75 | | 3.67 | | 2.81 | | 2.81 | | 0.31 | |  |
| Min Buri | 63.07 | 27.75 | 44.00 | 141,214 | 9,736 | 154.37 | 350.85 | 6.89 |  | 2.32 | | 34.81 | | 46.57 | | 6.70 | | 8.19 | | 0.72 | | 0.58 | | 0.12 | |  |
| Lat Krabang | 132.70 | 40.63 | 30.62 | 171,933 | 5,617 | 42.33 | 138.25 | 3.27 |  | 2.62 | | 41.32 | | 34.02 | | 14.69 | | 5.70 | | 0.69 | | 0.84 | | 0.12 | |  |
| Yannawa | 12.90 | 11.54 | 89.46 | 79,574 | 2,700 | 209.30 | 233.97 | 3.39 |  | 1.26 | | 26.78 | | 52.11 | | 7.89 | | 9.04 | | 1.67 | | 1.04 | | 0.22 | |  |
| Samphanthawong | 1.44 | 1.44 | 100.00 | 24,785 | 683 | 474.31 | 474.31 | 2.76 |  | 0.44 | | 23.87 | | 52.42 | | 7.17 | | 8.49 | | 3.66 | | 2.93 | | 1.02 | |  |
| Phaya Thai | 9.51 | 9.51 | 100.00 | 72,102 | 2,904 | 305.36 | 305.36 | 4.03 |  | 0.79 | | 21.38 | | 57.58 | | 7.33 | | 9.30 | | 1.72 | | 1.31 | | 0.59 | |  |
| Thon Buri | 8.73 | 8.73 | 100.00 | 111,027 | 3,018 | 345.70 | 345.70 | 2.72 |  | 1.52 | | 27.87 | | 54.74 | | 6.46 | | 4.71 | | 2.75 | | 1.62 | | 0.33 | |  |
| Bangkok Yai | 6.50 | 4.19 | 64.46 | 67,887 | 1,249 | 192.15 | 298.09 | 1.84 |  | 1.76 | | 21.78 | | 63.89 | | 4.32 | | 4.72 | | 1.84 | | 1.04 | | 0.64 | |  |
| Huai Khwang | 16.84 | 16.84 | 100.00 | 81,190 | 7,147 | 424.41 | 424.41 | 8.80 |  | 1.12 | | 25.44 | | 54.36 | | 7.68 | | 9.21 | | 1.01 | | 0.87 | | 0.32 | |  |
| Khlong San | 6.16 | 5.36 | 87.01 | 73,871 | 1,881 | 305.36 | 350.93 | 2.55 |  | 1.91 | | 27.11 | | 54.44 | | 6.91 | | 5.53 | | 2.82 | | 1.12 | | 0.16 | |  |
| Taling Chan | 36.92 | 20.80 | 56.34 | 105,289 | 3,473 | 94.07 | 166.97 | 3.30 |  | 1.44 | | 28.07 | | 53.21 | | 8.41 | | 7.08 | | 0.92 | | 0.75 | | 0.12 | |  |
| Bangkok Noi | 12.72 | 11.33 | 89.07 | 112,581 | 3,819 | 300.24 | 337.07 | 3.39 |  | 1.28 | | 24.51 | | 58.68 | | 5.92 | | 7.54 | | 0.86 | | 1.00 | | 0.21 | |  |
| Bang Khun Thian | 126.04 | 30.72 | 24.37 | 179,768 | 7,264 | 57.63 | 236.46 | 4.04 |  | 1.98 | | 35.35 | | 44.34 | | 7.90 | | 8.40 | | 0.98 | | 0.81 | | 0.23 | |  |
| Phasi Charoen | 19.79 | 13.46 | 68.01 | 259,418 | 5,968 | 301.57 | 443.39 | 2.30 |  | 1.93 | | 31.60 | | 52.83 | | 6.07 | | 5.19 | | 1.42 | | 0.72 | | 0.23 | |  |
| Nong Khaem | 37.35 | 22.42 | 60.03 | 155,229 | 8,745 | 234.14 | 390.05 | 5.63 |  | 3.18 | | 31.23 | | 51.39 | | 6.21 | | 6.38 | | 0.88 | | 0.59 | | 0.14 | |  |
| Rat Burana | 12.72 | 8.74 | 68.71 | 83,248 | 2,862 | 225.00 | 327.46 | 3.44 |  | 1.82 | | 31.62 | | 49.58 | | 6.81 | | 8.00 | | 1.05 | | 0.87 | | 0.24 | |  |
| Bang Phlat | 12.18 | 12.18 | 100.00 | 93,771 | 3,356 | 275.43 | 275.43 | 3.58 |  | 2.09 | | 24.52 | | 57.24 | | 6.62 | | 6.91 | | 1.19 | | 1.22 | | 0.21 | |  |
| Din Daeng | 8.74 | 8.74 | 100.00 | 123,966 | 6,378 | 729.75 | 729.75 | 5.14 |  | 1.22 | | 29.18 | | 49.80 | | 5.97 | | 10.44 | | 1.96 | | 1.11 | | 0.31 | |  |
| Bung Kum | 24.16 | 24.16 | 100.00 | 285,225 | 10,934 | 452.57 | 452.57 | 3.83 |  | 1.60 | | 30.34 | | 54.35 | | 5.51 | | 6.68 | | 0.95 | | 0.42 | | 0.15 | |  |
| Sa Thon | 7.49 | 7.49 | 100.00 | 80,497 | 3,145 | 419.89 | 419.89 | 3.91 |  | 1.02 | | 22.48 | | 59.46 | | 7.50 | | 5.98 | | 1.97 | | 1.21 | | 0.38 | |  |
| Bang Su | 13.30 | 9.34 | 70.23 | 126,136 | 2,935 | 220.68 | 314.28 | 2.33 |  | 1.26 | | 29.37 | | 52.98 | | 5.83 | | 8.89 | | 0.99 | | 0.55 | | 0.14 | |  |
| Chatuchak | 33.71 | 33.71 | 100.00 | 158,130 | 12,732 | 377.69 | 377.69 | 8.05 |  | 1.40 | | 25.79 | | 55.59 | | 7.29 | | 7.46 | | 1.33 | | 0.95 | | 0.20 | |  |
| Bang Kho Laem | 8.58 | 6.89 | 80.30 | 90,377 | 2,847 | 331.99 | 413.07 | 3.15 |  | 0.84 | | 25.50 | | 53.95 | | 7.20 | | 9.45 | | 1.62 | | 1.12 | | 0.32 | |  |
| Prawet | 55.42 | 31.97 | 57.69 | 294,501 | 6,967 | 125.70 | 217.93 | 2.37 |  | 2.01 | | 31.62 | | 47.06 | | 6.42 | | 11.31 | | 0.79 | | 0.66 | | 0.13 | |  |
| Khlong Toei | 13.66 | 11.53 | 84.41 | 188,739 | 3,018 | 220.94 | 261.65 | 1.60 |  | 1.03 | | 20.74 | | 57.42 | | 6.63 | | 10.11 | | 1.42 | | 2.12 | | 0.53 | |  |
| Suan Luang | 24.88 | 24.88 | 100.00 | 121,740 | 8,607 | 345.94 | 345.94 | 7.06 |  | 1.23 | | 29.60 | | 53.90 | | 5.47 | | 7.95 | | 0.90 | | 0.74 | | 0.22 | |  |
| Chom Thong | 23.90 | 16.26 | 68.03 | 208,214 | 8,126 | 339.97 | 499.65 | 3.90 |  | 1.26 | | 32.21 | | 53.45 | | 5.69 | | 5.33 | | 1.00 | | 0.90 | | 0.18 | |  |
| Don Mueang | 37.89 | 33.59 | 88.65 | 168,896 | 13,360 | 352.61 | 397.71 | 7.91 |  | 2.65 | | 30.15 | | 53.69 | | 4.72 | | 7.19 | | 0.91 | | 0.53 | | 0.16 | |  |
| Rat Thewi | 7.40 | 7.40 | 100.00 | 72,436 | 2,264 | 306.04 | 306.04 | 3.13 |  | 0.80 | | 23.10 | | 51.72 | | 9.36 | | 7.20 | | 4.02 | | 3.00 | | 0.80 | |  |
| Lat Phrao | 21.95 | 21.95 | 100.00 | 121,000 | 12,643 | 575.99 | 575.99 | 10.45 |  | 1.51 | | 24.57 | | 60.56 | | 4.82 | | 7.06 | | 0.66 | | 0.59 | | 0.21 | |  |
| Wattana | 13.41 | 13.41 | 100.00 | 84,528 | 4,271 | 318.49 | 318.49 | 5.05 |  | 0.87 | | 24.21 | | 54.17 | | 5.94 | | 10.87 | | 1.86 | | 1.42 | | 0.66 | |  |
| Bang Khae | 49.42 | 19.35 | 39.15 | 52,378 | 6,214 | 125.75 | 321.21 | 11.86 |  | 2.46 | | 32.97 | | 50.05 | | 6.29 | | 6.40 | | 0.82 | | 0.77 | | 0.23 | |  |
| Lak Si | 23.40 | 23.40 | 100.00 | 105,588 | 10,072 | 430.35 | 329.69 | 9.54 |  | 2.04 | | 27.11 | | 55.82 | | 5.37 | | 7.80 | | 0.91 | | 0.78 | | 0.16 | |  |
| Sai Mai | 45.03 | 25.30 | 56.18 | 200,374 | 10,717 | 238.00 | 423.54 | 5.35 |  | 3.44 | | 30.61 | | 49.44 | | 5.57 | | 9.50 | | 0.76 | | 0.50 | | 0.18 | |  |
| Khanna Yao | 26.19 | 26.19 | 100.00 | 49,575 | 8,780 | 335.19 | 333.74 | 17.71 |  | 1.66 | | 30.72 | | 51.99 | | 5.25 | | 9.00 | | 0.71 | | 0.51 | | 0.16 | |  |
| Saphan Sung | 28.96 | 22.86 | 78.94 | 94,982 | 7,990 | 275.90 | 349.52 | 8.41 |  | 2.25 | | 26.90 | | 60.32 | | 5.09 | | 4.35 | | 0.59 | | 0.36 | | 0.15 | |  |
| Wang Thong Lang | 17.62 | 17.62 | 100.00 | 112,849 | 8,948 | 507.80 | 507.80 | 7.93 |  | 1.30 | | 32.80 | | 46.67 | | 7.15 | | 10.27 | | 1.02 | | 0.61 | | 0.18 | |  |
| Khlong Sam Wa | 124.05 | 37.86 | 30.52 | 189,507 | 10,116 | 81.54 | 267.20 | 5.34 |  | 2.49 | | 36.47 | | 46.24 | | 7.13 | | 6.45 | | 0.64 | | 0.51 | | 0.07 | |  |
| Bang Na | 19.54 | 5.75 | 29.43 | 92,023 | 1,554 | 79.51 | 270.37 | 1.69 |  | 1.29 | | 32.82 | | 44.14 | | 9.91 | | 9.72 | | 0.97 | | 0.71 | | 0.45 | |  |
| Thawi Watthana | 53.40 | 25.33 | 47.43 | 77,890 | 4,207 | 78.79 | 166.10 | 5.40 |  | 2.21 | | 30.45 | | 44.69 | | 11.31 | | 10.27 | | 0.40 | | 0.52 | | 0.14 | |  |
| Thung Khu | 33.99 | 13.28 | 39.07 | 66,430 | 5,037 | 148.21 | 379.20 | 7.58 |  | 1.03 | | 34.70 | | 50.47 | | 5.06 | | 7.43 | | 0.66 | | 0.48 | | 0.18 | |  |
| Bang Bon | 36.99 | 23.84 | 64.45 | 107,136 | 9,568 | 258.66 | 401.38 | 8.93 |  | 1.71 | | 40.41 | | 44.25 | | 6.28 | | 5.43 | | 1.00 | | 0.77 | | 0.14 | |  |
| **Summary** | Total:  1,619.78 | Total:  870.56 | mean: 77.06 median: 88.65 SD: 26.14 | Total:  5,888,392 | Total:  298,391 | mean: 294.70 median: 301.57 SD: 159.46 | mean: 358.90 median: 345.94 SD: 119.79 | | mean: 5.30 median: 4.13 SD: 3.19 |  | Relative propotion over entire province (%): | | | | | | | | | | | | | | | |
|  |  |  |  |  |  |  |  |  |  |  | 1.78 | | 29.96 | | 51.84 | | 6.47 | | 7.82 | | 1.09 | | 0.81 | | 0.22 | |

**Table B.** Area, image coverage, population, and statistics of detected containers at the district level in Nakhon Si Thammarat, Thailand

| District Name | Area (km^2^) | Area of GSV images (km^2^) | GSV image coverage (%) | Population | Number of containers | Containers per km^2^ land | Containers per km^2^ image area | Containers per 100 population |  | Relative proportions of container types (%) | | | | | | | |
| --- | --- | --- | --- | --- | --- | --- | --- | --- | --- | --- | --- | --- | --- | --- | --- | --- | --- |
|  |  |  |  |  |  |  |  |  |  | Jar | Bucket | Potted plant | Tire | Bin | Bowl | Cup | Vase |
| Mueang Nakhon Si Thammarat | 564.61 | 111.50 | 19.70 | 271330 | 19915 | 35.27 | 178.62 | 7.34 |  | 1.78 | 41.91 | 36.77 | 6.30 | 11.77 | 0.57 | 0.81 | 0.10 |
| Phrom Khiri | 250.10 | 18.51 | 7.40 | 37513 | 2099 | 8.39 | 113.38 | 5.60 |  | 0.52 | 39.40 | 39.45 | 5.72 | 13.91 | 0.57 | 0.33 | 0.10 |
| Lan Saka | 349.54 | 15.24 | 4.40 | 40900 | 1479 | 4.23 | 97.02 | 3.62 |  | 2.10 | 46.79 | 31.58 | 5.07 | 13.79 | 0.34 | 0.27 | 0.07 |
| Chawang | 439.50 | 27.65 | 6.30 | 67293 | 3650 | 8.30 | 132.01 | 5.42 |  | 1.81 | 44.03 | 32.49 | 6.30 | 13.89 | 0.41 | 1.04 | 0.03 |
| Phipun | 501.18 | 11.86 | 2.40 | 29226 | 1812 | 3.62 | 152.74 | 6.20 |  | 1.77 | 51.27 | 32.62 | 6.07 | 6.40 | 0.72 | 0.77 | 0.39 |
| Chian Yai | 326.05 | 32.86 | 10.10 | 43457 | 2276 | 6.98 | 69.25 | 5.24 |  | 4.66 | 39.02 | 34.18 | 10.76 | 10.24 | 0.40 | 0.66 | 0.09 |
| Cha-uat | 760.30 | 53.01 | 7.00 | 86507 | 5335 | 7.02 | 100.65 | 6.17 |  | 1.61 | 51.73 | 27.39 | 9.99 | 7.48 | 0.88 | 0.81 | 0.11 |
| Tha Sala | 424.03 | 54.22 | 12.80 | 113067 | 4347 | 10.25 | 80.18 | 3.84 |  | 1.29 | 42.44 | 34.55 | 9.71 | 10.77 | 0.51 | 0.62 | 0.12 |
| Thung Song | 922.19 | 66.13 | 7.20 | 160724 | 8138 | 8.82 | 123.05 | 5.06 |  | 1.09 | 44.25 | 31.13 | 10.72 | 11.53 | 0.53 | 0.71 | 0.05 |
| Na Bon | 210.68 | 15.23 | 7.20 | 26934 | 1986 | 9.43 | 130.42 | 7.37 |  | 0.96 | 58.61 | 21.25 | 8.61 | 9.62 | 0.35 | 0.60 | 0.00 |
| Thung Yai | 609.19 | 47.75 | 7.80 | 74317 | 5781 | 9.49 | 121.06 | 7.78 |  | 1.35 | 65.91 | 19.49 | 5.09 | 6.87 | 0.42 | 0.86 | 0.02 |
| Pak Phanang | 537.92 | 73.37 | 13.60 | 130160 | 6546 | 12.17 | 89.22 | 5.03 |  | 6.29 | 45.88 | 30.78 | 8.62 | 6.94 | 0.75 | 0.63 | 0.12 |
| Ron Phibun | 435.26 | 24.65 | 5.70 | 82031 | 1501 | 3.45 | 60.90 | 1.83 |  | 3.60 | 27.85 | 43.84 | 13.26 | 10.26 | 0.73 | 0.33 | 0.13 |
| Sichon | 698.17 | 50.94 | 7.30 | 88611 | 3052 | 4.37 | 59.92 | 3.44 |  | 2.92 | 28.21 | 33.91 | 10.94 | 23.03 | 0.43 | 0.46 | 0.10 |
| Khanom | 315.83 | 24.28 | 7.70 | 30393 | 2364 | 7.49 | 97.35 | 7.78 |  | 2.33 | 55.54 | 26.90 | 7.19 | 7.28 | 0.38 | 0.34 | 0.04 |
| Hua Sai | 442.98 | 44.31 | 10.00 | 66503 | 2881 | 6.50 | 65.03 | 4.33 |  | 4.37 | 48.14 | 32.87 | 8.82 | 4.30 | 0.62 | 0.80 | 0.07 |
| Bang Khan | 475.96 | 29.72 | 6.20 | 46914 | 1875 | 3.94 | 63.09 | 4.00 |  | 7.25 | 38.93 | 36.48 | 12.96 | 3.36 | 0.16 | 0.59 | 0.27 |
| Tham Phannara | 179.00 | 10.15 | 5.70 | 19177 | 889 | 4.97 | 87.62 | 4.64 |  | 2.02 | 41.17 | 26.32 | 21.48 | 8.10 | 0.67 | 0.22 | 0.00 |
| Chulabhorn | 233.58 | 16.14 | 6.90 | 31584 | 2231 | 9.55 | 138.20 | 7.06 |  | 1.08 | 37.38 | 29.54 | 18.69 | 12.01 | 0.54 | 0.76 | 0.00 |
| Phra Phrom | 151.07 | 29.95 | 19.80 | 43588 | 2880 | 19.06 | 96.15 | 6.61 |  | 2.95 | 48.19 | 30.21 | 10.35 | 7.26 | 0.35 | 0.59 | 0.10 |
| Nopphitam | 732.73 | 18.68 | 2.50 | 33320 | 1293 | 1.76 | 69.22 | 3.88 |  | 2.17 | 33.02 | 34.42 | 9.44 | 18.95 | 1.39 | 0.54 | 0.08 |
| Chang Klang | 276.62 | 10.90 | 3.90 | 29909 | 839 | 3.03 | 76.97 | 2.81 |  | 1.55 | 61.98 | 25.15 | 5.24 | 4.53 | 0.60 | 0.95 | 0.00 |
| Chaloem Phra Kiet | 183.34 | 21.09 | 11.50 | 31572 | 1440 | 7.85 | 68.29 | 4.56 |  | 6.53 | 33.33 | 36.94 | 18.26 | 3.47 | 0.76 | 0.56 | 0.14 |
| **Summary** | Total: 10,019.83 | Total: 808.14 | mean: 8.40 median: 7.20 SD: 4.57 | Total: 1,585,030 | Total: 84,609 | mean: 8.52 median: 7.49 SD: 6.91 | mean: 98.71 median: 96.15 SD: 32.56 | mean: 5.20 median: 5.06 SD: 1.64 |  | Relative proportion over entire province (%): | | | | | | | |
|  |  |  |  |  |  |  |  |  |  | 2.44 | 45.14 | 32.08 | 8.78 | 10.21 | 0.56 | 0.70 | 0.09 |

**Table C.** Area, image coverage, population, and statistics of detected containers at the district level in Krabi, Thailand

| District Name | Area (km^2^) | Area of image (km^2^) | image coverage (%) | Population | Number of containers | Containers per km^2^ land | Containers per km^2^ image area | Containers per 100 population |  | Relative proportions of container types (%) | | | | | | | |
| --- | --- | --- | --- | --- | --- | --- | --- | --- | --- | --- | --- | --- | --- | --- | --- | --- | --- |
|  |  |  |  |  |  |  |  |  |  | Jar | Bucket | Potted plant | Tire | Bin | Bowl | Cup | Vase |
| Mueang Krabi | 887.13 | 52.48 | 5.92 | 118288 | 2597 | 2.93 | 49.48 | 2.20 |  | 3.20 | 46.17 | 34.39 | 10.47 | 3.97 | 0.69 | 0.85 | 0.27 |
| Khao Phanom | 861.73 | 44.41 | 5.15 | 54956 | 3367 | 3.91 | 75.81 | 6.13 |  | 4.34 | 47.46 | 30.35 | 8.64 | 8.32 | 0.12 | 0.71 | 0.06 |
| Ko Lanta | 249.33 | 18.85 | 7.56 | 34337 | 1471 | 5.90 | 78.03 | 4.28 |  | 1.70 | 61.52 | 24.81 | 6.59 | 3.20 | 0.61 | 1.43 | 0.14 |
| Khlong Thom | 1132.32 | 70.66 | 6.24 | 76798 | 5339 | 4.72 | 75.56 | 6.95 |  | 2.53 | 53.81 | 25.75 | 10.62 | 5.84 | 0.49 | 0.82 | 0.13 |
| Ao Luek | 801.07 | 58.98 | 7.36 | 56138 | 5587 | 6.97 | 94.73 | 9.95 |  | 3.11 | 48.31 | 26.69 | 9.68 | 10.90 | 0.34 | 0.84 | 0.13 |
| Plai Phraya | 536.93 | 42.75 | 7.96 | 38578 | 2803 | 5.22 | 65.57 | 7.27 |  | 4.50 | 49.30 | 30.07 | 6.35 | 8.46 | 0.36 | 0.71 | 0.25 |
| Lam Thap | 285.40 | 19.75 | 6.92 | 24202 | 2367 | 8.29 | 119.83 | 9.78 |  | 2.83 | 47.36 | 31.77 | 8.37 | 8.45 | 0.55 | 0.51 | 0.17 |
| Nuea Khlong | 479.82 | 54.53 | 11.36 | 62634 | 6494 | 13.53 | 119.09 | 10.37 |  | 1.45 | 60.32 | 23.61 | 7.13 | 5.99 | 0.48 | 0.95 | 0.08 |
| **Summary** | Total: 5,233.73 | Total: 362.41 | mean: 7.31 median: 7.14 SD: 1.88 | Total: 465,931 | Total: 30,025 | mean: 6.43 median: 5.56 SD: 3.33 | mean: 84.76 median: 76.92 SD: 24.87 | mean: 7.12 median: 7.11 SD: 2.90 |  | Relative proportion over entire province (%): | | | | | | | |
|  |  |  |  |  |  |  |  |  |  | 2.83 | 52.27 | 27.56 | 8.68 | 7.25 | 0.43 | 0.84 | 0.14 |

**Table D.** Statistics of detected containers for sub-districts where BI values were collected during the dengue season.

| District Name | Subdistrict Name | Area (km^2^) | Area of image (km^2^) | image coverage (%) | Population | Number of containers | Containers per km^2^ land | Containers per km^2^ image area | Breeding site per 100 population |  | Relative proportion of BS types (%) | | | | | | | |
| --- | --- | --- | --- | --- | --- | --- | --- | --- | --- | --- | --- | --- | --- | --- | --- | --- | --- | --- |
|  |  |  |  |  |  |  |  |  |  |  | Jar | Bucket | Potted plant | Tire | Bin | Bowl | Cup | Vase |
| Mueang Nakhon Si Thammarat | Kamphaeng Sao | 31.00 | 4.49 | 14.47 | 9559 | 865 | 27.90 | 192.65 | 9.05 |  | 0.12 | 56.65 | 21.73 | 4.86 | 15.84 | 0.23 | 0.46 | 0.12 |
| Mueang Nakhon Si Thammarat | Chai Montri | 14.87 | 3.78 | 25.42 | 6544 | 658 | 44.25 | 174.07 | 10.06 |  | 1.06 | 65.81 | 20.67 | 5.17 | 6.84 | 0.00 | 0.46 | 0.00 |
| Mueang Nakhon Si Thammarat | Mamuang Song Ton | 7.89 | 2.10 | 26.64 | 5013 | 313 | 39.67 | 149.05 | 6.24 |  | 5.11 | 27.80 | 40.58 | 8.31 | 17.57 | 0.64 | 0.00 | 0.00 |
| Chawang | Na Wae | 36.61 | 3.23 | 8.83 | 7372 | 472 | 12.89 | 146.13 | 6.40 |  | 1.69 | 51.48 | 30.93 | 7.63 | 6.14 | 0.42 | 1.69 | 0.00 |
| Chawang | Huai Prik | 66.31 | 3.23 | 4.87 | 6512 | 434 | 6.55 | 134.37 | 6.66 |  | 1.84 | 52.76 | 26.27 | 6.45 | 11.06 | 0.23 | 1.38 | 0.00 |
| Chawang | Na Khliang | 19.94 | 1.16 | 5.82 | 2845 | 278 | 13.94 | 239.66 | 9.77 |  | 2.16 | 39.21 | 27.70 | 6.12 | 23.38 | 0.00 | 1.44 | 0.00 |
| Phipun | Kathun | 121.62 | 3.62 | 2.98 | 5540 | 483 | 3.97 | 133.43 | 8.72 |  | 2.07 | 49.28 | 33.13 | 6.63 | 5.38 | 2.28 | 0.83 | 0.41 |
| Cha-uat | Cha-Uat | 72.46 | 8.24 | 11.37 | 12683 | 639 | 8.82 | 77.55 | 5.04 |  | 2.19 | 51.49 | 31.30 | 7.20 | 5.79 | 0.78 | 1.10 | 0.16 |
| Cha-uat | Tha Pracha | 27.56 | 3.71 | 13.46 | 7227 | 426 | 15.46 | 114.82 | 5.89 |  | 1.88 | 30.52 | 30.52 | 7.28 | 26.29 | 1.64 | 1.64 | 0.23 |
| Cha-uat | Wang Ang | 126.69 | 3.73 | 2.95 | 10304 | 531 | 4.19 | 142.36 | 5.15 |  | 0.56 | 33.52 | 27.50 | 21.28 | 14.31 | 1.13 | 1.69 | 0.00 |
| Cha-uat | Ban Tun | 82.70 | 6.57 | 7.95 | 7133 | 340 | 4.11 | 51.75 | 4.77 |  | 2.65 | 50.00 | 35.88 | 9.41 | 1.18 | 0.29 | 0.59 | 0.00 |
| Cha-uat | Khon Hat | 60.45 | 5.27 | 8.72 | 5908 | 423 | 7.00 | 80.27 | 7.16 |  | 1.89 | 63.36 | 24.82 | 6.86 | 0.71 | 1.18 | 0.95 | 0.24 |
| Cha-uat | Ko Khan | 42.53 | 3.52 | 8.28 | 8989 | 153 | 3.60 | 43.47 | 1.70 |  | 0.00 | 33.33 | 29.41 | 26.14 | 9.80 | 1.31 | 0.00 | 0.00 |
| Cha-uat | Khuan Nong Hong | 39.78 | 5.36 | 13.48 | 7327 | 766 | 19.26 | 142.91 | 10.45 |  | 1.96 | 42.69 | 26.24 | 17.10 | 10.57 | 0.78 | 0.65 | 0.00 |
| Cha-uat | Khao Phra Thong | 53.88 | 2.78 | 5.17 | 8152 | 153 | 2.84 | 55.04 | 1.88 |  | 0.00 | 33.33 | 29.41 | 26.14 | 9.80 | 1.31 | 0.00 | 0.00 |
| Thung Song | Nong Hong | 27.05 | 3.04 | 11.25 | 11010 | 470 | 17.38 | 154.61 | 4.27 |  | 0.64 | 34.89 | 27.23 | 12.34 | 23.83 | 0.43 | 0.64 | 0.00 |
| Thung Song | Khao Ro | 88.96 | 7.02 | 7.89 | 10575 | 244 | 2.74 | 34.76 | 2.31 |  | 2.05 | 18.85 | 65.16 | 10.66 | 2.87 | 0.00 | 0.41 | 0.00 |
| Thung Song | Thi Wang | 79.99 | 5.40 | 6.75 | 13912 | 723 | 9.04 | 133.89 | 5.20 |  | 0.55 | 65.42 | 20.06 | 10.37 | 2.90 | 0.14 | 0.55 | 0.00 |
| Thung Song | Na Pho | 30.89 | 4.45 | 14.42 | 6304 | 621 | 20.10 | 139.55 | 9.85 |  | 1.61 | 31.56 | 28.50 | 12.24 | 24.80 | 0.64 | 0.64 | 0.00 |
| Na Bon | Kaeo Saen | 68.30 | 6.73 | 9.85 | 7125 | 504 | 7.38 | 74.89 | 7.07 |  | 0.60 | 47.02 | 26.98 | 13.29 | 10.52 | 0.99 | 0.60 | 0.00 |
| Thung Yai | Thung Sang | 63.14 | 7.05 | 11.16 | 5620 | 510 | 8.08 | 72.34 | 9.07 |  | 1.57 | 41.76 | 24.51 | 4.71 | 25.10 | 0.78 | 1.57 | 0.00 |
| Thung Yai | Kurae | 84.83 | 5.97 | 7.04 | 8142 | 757 | 8.92 | 126.80 | 9.30 |  | 1.45 | 73.98 | 18.49 | 3.30 | 1.98 | 0.26 | 0.53 | 0.00 |
| Thung Yai | Krung Yan | 115.96 | 7.27 | 6.27 | 10583 | 903 | 7.79 | 124.21 | 8.53 |  | 2.10 | 73.53 | 18.05 | 2.33 | 2.88 | 0.44 | 0.66 | 0.00 |
| Bang Khan | Bang Khan | 158.87 | 9.90 | 6.23 | 13973 | 433 | 2.73 | 43.74 | 3.10 |  | 8.78 | 28.87 | 43.42 | 13.16 | 4.85 | 0.23 | 0.69 | 0.00 |
| Bang Khan | Ban Lamnao | 168.00 | 8.85 | 5.27 | 16284 | 918 | 5.46 | 103.73 | 5.64 |  | 6.10 | 49.46 | 31.05 | 9.80 | 2.61 | 0.11 | 0.54 | 0.33 |
| Bang Khan | Wang Hin | 76.25 | 6.28 | 8.24 | 9002 | 327 | 4.29 | 52.07 | 3.63 |  | 4.89 | 26.30 | 39.76 | 23.24 | 4.59 | 0.31 | 0.61 | 0.31 |
| Bang Khan | Ban Nikhom | 72.84 | 4.69 | 6.44 | 7655 | 197 | 2.70 | 42.00 | 2.57 |  | 13.20 | 32.99 | 41.12 | 10.15 | 1.52 | 0.00 | 0.51 | 0.51 |
| Chulabhorn | Thung Pho | 74.07 | 6.50 | 8.78 | 9776 | 919 | 12.41 | 141.38 | 9.40 |  | 1.09 | 42.00 | 26.22 | 13.60 | 15.78 | 0.44 | 0.87 | 0.00 |
| Chulabhorn | Na Mo Bun | 59.54 | 3.89 | 6.53 | 7146 | 527 | 8.85 | 135.48 | 7.37 |  | 1.14 | 36.05 | 30.55 | 21.25 | 9.49 | 0.76 | 0.76 | 0.00 |
| **Summary** |  | Total: 1,972.98 | Total: 147.83 | mean: 9.54 median: 8.24 SD: 5.53 | Total: 248,215 | Total: 14,987 | mean: 11.46 med: 8.08 SD: 10.46 | mean: 112.31 med: 126.80 SD: 50.98 | mean: 6.42 med: 6.40 SD: 2.69 |  | Relative proportion over 29 sub-districts (%): | | | | | | | |
|  |  |  |  |  |  |  |  |  |  |  | 2.31 | 47.46 | 28.42 | 10.53 | 9.81 | 0.59 | 0.81 | 0.07 |

**Table E.** Statistics for detected containers in sub-districts of Lansaka district of Nakhon Si Thammarat.

| District Name | Subdistrict Name | Area (km^2^) | Area of image (km^2^) | Image coverage (%) | Population | Number of containers | Containers per km^2^ land | Containers per km^2^ image area | Containers per 100 pop |  | Relative proportion of container types (%) | | | | | | | |
| --- | --- | --- | --- | --- | --- | --- | --- | --- | --- | --- | --- | --- | --- | --- | --- | --- | --- | --- |
|  |  |  |  |  |  |  |  |  |  |  | Jar | Bucket | Potted plant | Tire | Bin | Bowl | Cup | Vase |
| Lan Saka | Khao Kaeo | 86.48 | 1.20 | 1.39 | 7620 | 24 | 0.28 | 20.00 | 0.31 |  | 0.00 | 45.83 | 37.50 | 4.17 | 12.50 | 0.00 | 0.00 | 0.00 |
| Lan Saka | Kamlon | 96.43 | 2.72 | 2.82 | 9125 | 318 | 3.30 | 116.91 | 3.48 |  | 1.89 | 46.23 | 37.11 | 3.46 | 10.06 | 0.63 | 0.31 | 0.31 |
| Lan Saka | Tha Di | 35.49 | 2.56 | 7.21 | 7974 | 445 | 12.54 | 173.83 | 5.58 |  | 0.90 | 52.58 | 28.09 | 4.27 | 13.71 | 0.22 | 0.22 | 0.00 |
| Lan Saka | Khun Thale | 51.88 | 6.07 | 11.70 | 10296 | 446 | 8.60 | 73.48 | 4.33 |  | 3.59 | 47.76 | 30.04 | 8.74 | 8.97 | 0.45 | 0.45 | 0.00 |
| Lan Saka | Lan Saka | 79.27 | 2.69 | 3.39 | 5885 | 246 | 3.10 | 91.45 | 4.18 |  | 2.03 | 35.37 | 32.93 | 2.03 | 27.64 | 0.00 | 0.00 | 0.00 |
| **Summary** |  | Total: 349.54 | Total: 15.24 | mean: 5.30 median: 3.39 SD: 4.17 | Total:  40,900 | Total:  1,479 | mean: 5.56 median: 3.30 SD: 4.92 | mean: 95.13 median: 91.45 SD: 56.56 | mean: 3.58 median: 4.18 SD: 1.98 |  | Relative proportion over entire district (%): | | | | | | | |
|  |  |  |  |  |  |  |  |  |  |  | 2.10 | 46.79 | 31.58 | 5.07 | 13.79 | 0.34 | 0.27 | 0.07 |
